# The graphite-catalyzed *ipso*-functionalization of arylboronic acids in an aqueous medium: metal-free access to phenols, anilines, nitroarenes, and haloarenes†

**DOI:** 10.1039/d1ra01940f

**Published:** 2021-05-19

**Authors:** Anshu Dandia, Ruchi Sharma, Pratibha Saini, Ranveer Singh Badgoti, Kuldeep S. Rathore, Vijay Parewa

**Affiliations:** Centre of Advanced Studies, Department of Chemistry, University of Rajasthan Jaipur India dranshudandia@yahoo.co.in parewavijay.parewa@gmail.com parewavijay@uniraj.ac.in; Department of Physics, Arya College of Engineering and IT Jaipur India kuldeep_ssr@yahoo.com

## Abstract

An efficient, metal-free, and sustainable strategy has been described for the *ipso*-functionalization of phenylboronic acids using air as an oxidant in an aqueous medium. A range of carbon materials has been tested as carbocatalysts. To our surprise, graphite was found to be the best catalyst in terms of the turnover frequency. A broad range of valuable substituted aromatic compounds, *i.e.*, phenols, anilines, nitroarenes, and haloarenes, has been prepared *via* the functionalization of the C–B bond into C–N, C–O, and many other C–X bonds. The vital role of the aromatic π-conjugation system of graphite in this protocol has been established and was observed *via* numerous analytic techniques. The heterogeneous nature of graphite facilitates the high recyclability of the carbocatalyst. This effective and easy system provides a multipurpose approach for the production of valuable substituted aromatic compounds without using any metals, ligands, bases, or harsh oxidants.

## Abstract 2

An efficient, metal-free, and sustainable strategy has been described for the *ipso*-functionalization of phenylboronic acids using air as an oxidant in an aqueous medium.

## Graphical abstract

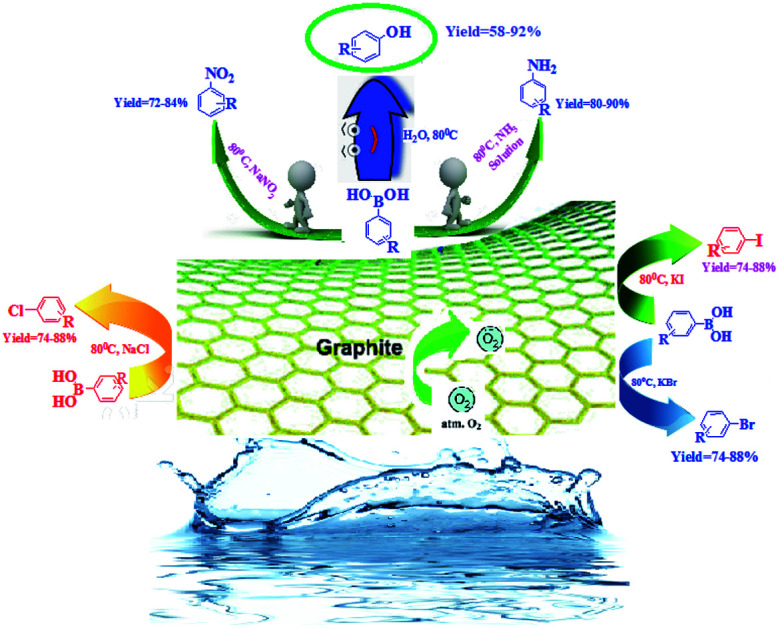

## 1. Introduction

Arylboronic acids and their derivatives have attracted substantial research interest from chemists since they have immense value in chemical synthesis and materials science.^1^ They have also been used as synthons for the further construction of therapeutic molecules and value-added chemicals.^2^ Furthermore, arylboronic acids have been extensively used as ubiquitous structural motifs for various functional group transformations *via ipso*-functionalization.^3^

Phenols are a well-known class of compounds for their biological applications, including antibacterial, antitumor, antiviral, pro-oxidant, cardioprotective, and antimutagenicity activities.^4^ They have also been employed as synthetic intermediates in various chemical transformations.^5^ Due to the wide-ranging applications of phenols, dedicated efforts have been made to advance the preparation of phenols.^6^ Among them, the *ipso*-hydroxylation of arylboronic acid has been the most-studied strategy in the recent past.^7–9^

This transformation is usually carried out with several equivalents of an oxidant, like hypervalent iodine, *tert*-butyl hydroperoxide (TBHP), ozone, PhI(OAc)_2_, benzoquinone, or hydrogen peroxide (H_2_O_2_), which can be explosive or have a hazardous environmental impact. These reactions can also entail high temperatures, strongly basic conditions, and the use of metal catalysts (Pd, Au, Cu, Ru, Ag, *etc.*) and microwave irradiation, and they often require the use of expensive specialized ligands. As phenols are susceptible to oxidants, the quantity of oxidant needs to be cautiously determined. Moreover, the removal of trace quantities of metal-containing catalysts from the final compounds can be complicated. Therefore, the development of sustainable protocols for the above transformation presents a difficult task for chemists.

To meet the requirements of green chemistry, carbon-based materials, such as ordered mesoporous carbon, activated carbon, graphene-based materials, graphite, and carbon nanotubes, open up opportunities in the field of catalysis due to their inimitable physical and chemical properties.^10^ In recent years, many scientists have established that carbon-based materials can show elevated catalytic efficiencies in organic synthesis compared to traditional metal catalysts.^11^ The sustainable nature of these carbon-based materials has been well documented in the literature.^12^ Among them, the use of graphite as an environmentally benign catalyst in organic synthesis has taken on vast significance.^13^

**Scheme 1:**
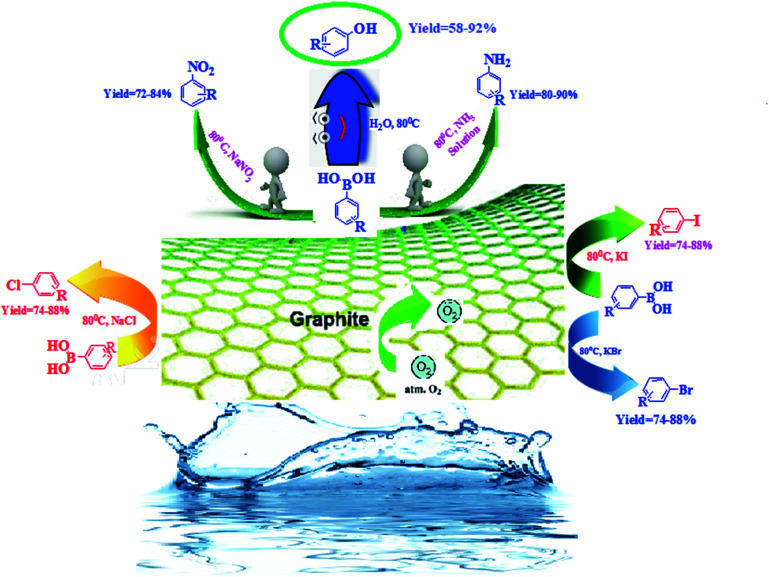
The synthesis of substituted phenols, anilines, nitroarenes, and haloarenes from arylboronic acids.

Continuing our interest in developing sustainable protocols for organic synthesis,^14^ herein, we evaluate the catalytic activities of various carbon-based substances for the preparation of a library of phenol derivatives *via* the *ipso*-hydroxylation of arylboronic acid, utilizing water as an environmentally friendly solvent under sustainable conditions. Commercially available graphite was found to be the best metal-free carbocatalyst for the above transformation. Furthermore, we have also extended our strategy to the synthesis of anilines, nitroarenes, and haloarenes *via* transforming the C–B bond of arylboronic acid to C–N and many other C–X bonds (Scheme 1).

## 2. Results and discussion

Firstly, phenylboronic acid has been selected as a model reactant for *ipso*-hydroxylation in an aqueous medium under air at 80 °C (Table 1). No transformation of phenylboronic acid into phenol was noticed in the absence of catalyst. This outcome forced us to further optimize the reaction conditions. Recently, we have developed a series of carbon-based catalysts for various organic transformations.^15^ Therefore, from a sustainable point of view, the catalytic competences of various carbocatalysts have been studied. To our surprise, graphite was found to be the best catalyst for this transformation in terms of the turnover frequency (TOF^15*b*,16^). Moreover, inferior yields were observed upon using other carbon-based materials as catalysts. GO and rGO gave poor product yields. Approximately similar catalytic activities were observed when the reaction was tried with various graphite samples purchased from different vendors.

**Table 1:** The catalytic competences of various carbocatalysts for the *ipso*-hydroxylation of phenylboronic acid^a^

S. no.	Catalyst	Solvent	Time	Yield^b^ (%)	TOF (×10^−3^ mol g^−1^ min^−1^)
1	—	H_2_O	24 h	—	n.c.
2	Graphite (20 wt%)	H_2_O	24 h	92	0.021
3	Activated carbon (20 wt%)	H_2_O	24 h	74	0.017
4	MWCNTs (20 wt%)^c^	H_2_O	24 h	72	0.016
5	GO (20 wt%)	H_2_O	24 h	21	0.005
6	rGO (20 wt%)	H_2_O	24 h	48	0.011
7	Graphite (5 wt%)	H_2_O	24 h	64	0.069
8	Graphite (10 wt%)	H_2_O	24 h	79	0.041
9	Graphite (20 wt%)^d^	H_2_O	24 h	—	—
10	Graphite (20 wt%)	CH_3_CN	24 h	82	0.019
11	Graphite (20 wt%)	MeOH	24 h	69	0.016
12	Graphite (20 wt%)	THF	24 h	44	0.010
13	Graphite (20 wt%)	DMSO	24 h	38	0.009

a Reactions were carried out with phenylboronic acid (2 mmol) in H_2_O at 80 °C under air.

b Isolated yield.

c Multi-walled carbon nanotubes.

d Conversion conducted under a N_2_ atmosphere.

An improvement in the yield of the desired product to an immense extent (36–92%) was observed when the catalyst loading was increased from 5 to 20 wt% (Table 1). We have tried different catalyst loadings (20, 30, 40, and 50 wt%), however further increases in catalyst loading did not affect the yield, and the optimum loading of catalyst was 20 wt%. The desired product was not formed when the model reaction was conducted under a nitrogen atmosphere. These results show that, from a sustainable point of view, air is an effective and environmentally benign oxidant. The desirable transformation was not observed in the absence of graphite, which shows that graphite plays a vital role in the further activation of oxygen molecules from air for the catalytic reaction.

A series of trials was carried out using a variety of solvents. Methanol and acetonitrile were found to give moderate yields among the various organic solvents. Compared to conventional organic solvents, H_2_O was found to be a best solvent for the above transformation from an economical perspective. The outcome can be attributed to the excellent dispersion of the catalyst in H_2_O. Additionally, the substrates show good dissolving capacities in water. The model reaction was also performed at different temperatures (60 °C, 80 °C, and 100 °C). It was concluded that, to achieve maximum conversion to the product, 80 °C was required.

To recognize the responsible catalytic sites in carbon-based catalysts, various organic compounds were employed as catalysts for the above transformation. Compounds with various oxygen functionalities showed poor catalytic activities. These outcomes confirmed that oxygen-containing functional groups may not be the catalytic sites in the carbon materials for hydroxylation. It was recognized from various catalytic tests that compounds containing aromatic π-conjugation systems demonstrated reasonable catalytic activities (Table 2). We suppose that the aromatic π-conjugation system has intrinsic activity for the above transformation. Thus, carbon materials that have significant oxygen functionality showed poor results compared to graphite.

**Table 2:** The catalytic activities of various organic counterparts^a^

S. no.	Catalyst	Time	Yield^b^ (%)
1	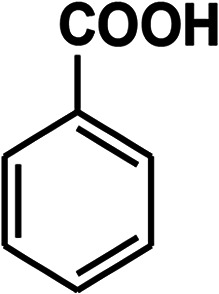	72 h	8
2	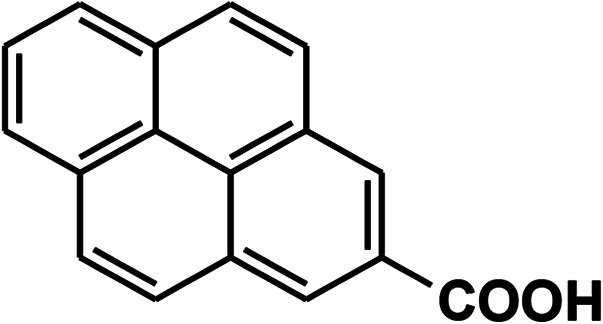	72 h	29
3	HCOOH	72 h	—
4	CH_3_COOH	72 h	—
5	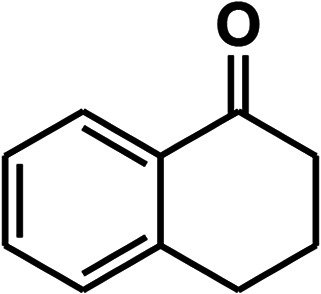	72 h	4
6	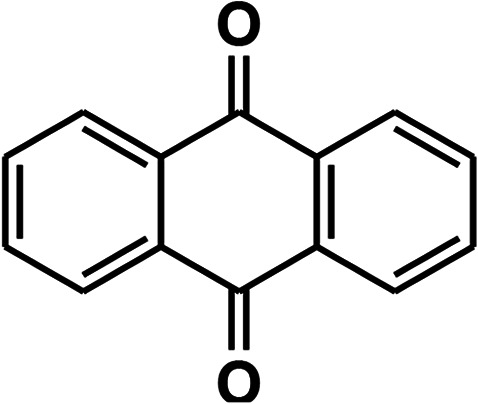	72 h	12
7	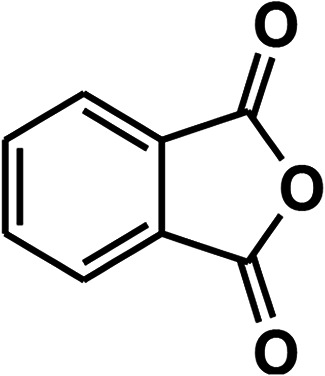	72 h	7
8	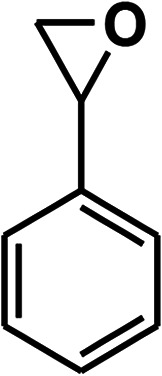	72 h	5
9	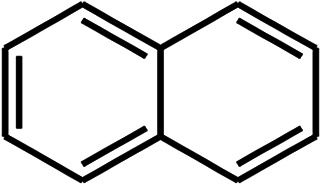	24 h	42
10	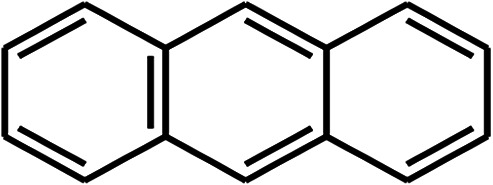	24 h	58

a Reactions were carried out with phenylboronic acid (2 mmol) in H_2_O at 80 °C under air.

b Isolated yield.

Carbon materials illustrate metallic features and activate O_2_ due to the presence of delocalized π-electrons.^17^ The introduction of any surface defects into a carbon material decreases its catalytic activity because this diminishes the mobility of delocalized π-electrons.^18^ Activated charcoal showed lower catalytic activity due to its decreased mass transfer efficiency because of its high degree of defects and its microporous structure.^19^ It has been revealed in the literature that sp^2^-hybridized carbon atoms are vital centers for oxygen adsorption and activation.^17^ These activated oxygen atoms can move on graphitic planes. As we already showed the necessity of oxygen for the above transformation, we proposed that the activation of oxygen was a decisive step in this transformation. The graphitic planes of the catalyst transform atmospheric O_2_ to short-lived O_2_˙^−^ species that are stored in the carbon pores.^17^

There is a possibility that the observed catalytic properties of graphite could be attributed to the presence of trace metal-ion impurities. To establish the presence of trace amounts of metal ions, we have studied graphite catalyst samples (fresh and reused) *via* various analytical techniques. EDAX analysis confirmed the absence of any transition metals in the graphite catalyst, which ruled out catalytic assistance from any metal impurities. Moreover, the carbon : oxygen atomic ratios of fresh and reused graphite were comparable, showing the stable nature of the graphite catalyst during the reaction (see ESI, Fig. S1 and S2†).

**Fig. 1:**
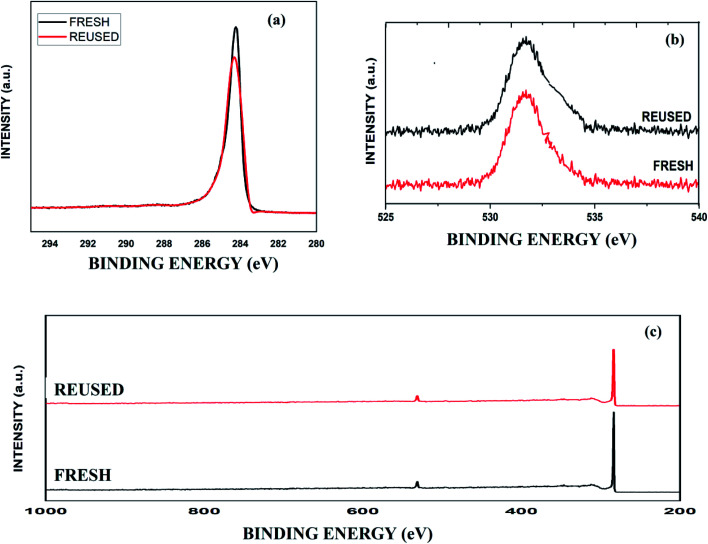
The XPS spectra of fresh and reused graphite: (a) C 1s spectra; (b) O 1s spectra; and (c) the complete XPS spectra.

The deconvoluted C 1s XPS spectrum of fresh graphite shows a peak at 284.4 eV. Additionally, the O 1s spectrum illustrates a broad peak from 530 to 535 eV. The intensities and energies of the peaks of reused graphite in both XPS spectra were approximately the same (Fig. 1).

**Fig. 2:**
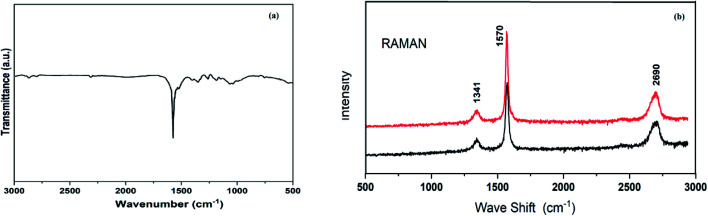
(a) The FT-IR spectrum of reused graphite and (b) Raman spectra of fresh and reused graphite.

In the Fourier-transform infrared (FT-IR) spectrum of reused graphite, absorption peak fluctuations from 1443 to 1573 cm^−1^ are noted because of aromatic C=C stretching (Fig. 2a). We have also carried out solid-state ^13^C-NMR spectroscopy analysis of reused graphite (see ESI, Fig. S5†). The spectrum showed broad resonance associated with sp^2^-hybridized carbon atoms.^20^ There were no additional peaks from any oxygen functionalities in the FT-IR and solid-state ^13^C-NMR spectra of reused graphite.

The Raman spectra of fresh and reused catalyst graphite samples comprise many overlapping bands (Fig. 2b). The D band, G band, and 2D band arise at around 1341 cm^−1^, 1570 cm^−1^, and 2690 cm^−1^, respectively.

**Fig. 3:**
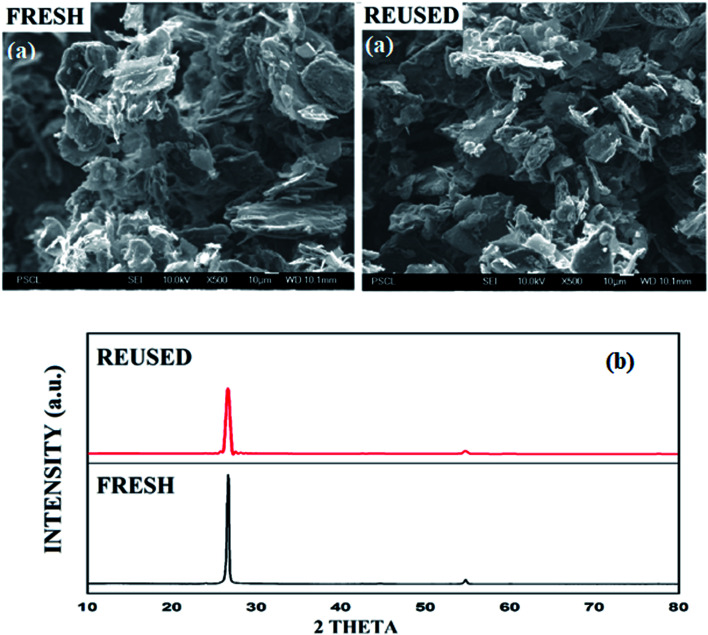
(a) SEM images and (b) XRD patterns of fresh and reused graphite.

In SEM images, the graphite sheets displayed crumpled and wrinkled surfaces of thin lamellae (Fig. 3a, see ESI, Fig. S3†). The average particle size of the catalyst is about 9.8 μm. The morphology and average particle size of the reused catalyst remain unchanged, as shown by SEM images of the reused graphite catalyst after four runs. The XRD pattern of recycled graphite was similarly documented, indicating that the structural integrity remains unchanged after the transformation reaction (Fig. 3b).

These analytical results proved that no oxidation of the catalyst was observed during the reaction, and the aromatic nature of the catalyst remains constant throughout the process. These results demonstrate that the utilized graphite catalyst is a stable and reusable catalyst for the above transformation.

To further confirm the role of the catalyst in the activation of the oxygen, EPR measurements were carried out with DMPO (see ESI, Fig. S5†). When the EPR experiment was performed with catalyst, peaks from the spin adduct of the DMPO superoxide radical were observed,^21^ while in the absence of catalyst, these peaks were absent. This result indicated that the generation of superoxide radicals was indispensable for the reaction to proceed.

**Scheme 2:**
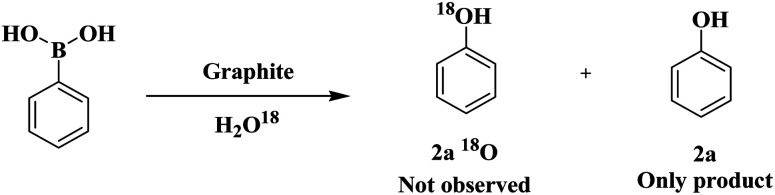
Isotopic experiment results.

These results suggest that a radical process might be involved in the above transformation. We also conducted the reaction with H_2_O^18^ as a solvent under optimized conditions. The incorporation of ^18^O into the product was not viewed, which ruled out the possibility of water acting as an oxygen source (Scheme 2).

In most cases, literature reports reveal the requirement for a base/metal in the reaction when phenylboronic acid reacts with reactive oxygen species.^22^ The mechanism of the reaction is ambiguous, particularly in light of the fact that the reaction can occur in the absence of any base/metal under air. *Via* control experiments, we have already confirmed that a radical process might be involved in the transformation. Due to the presence of delocalized π-electrons, graphitic planes convert O_2_ to short-lived O_2_˙^−^ species that assemble in the carbon pores.^17^ This short-lived O_2_˙^−^ was the active sustainable oxidant for the above transformation. Based on the above information and literature reports,^23^ we considered that the reaction occurred *via* a radical nucleophilic substitution mechanism^24^ through an unexplored process (to be the subject of future investigations). Owing to the presence of a delocalized π-electron system, graphite also stabilizes the radical intermediates for better reaction performance.

To elaborate on the possibilities of this approach, numerous electronically diversified phenylboronic acids were used for hydroxylation under the established optimum conditions (Table 3). This approach shows broad functional-group tolerance. Arylboronic acids containing electron-withdrawing and -donating substituents at the *para* position gave excellent phenol yields. The use of sterically demanding *ortho*-substituted arylboronic acids did not influence the yields drastically, and the final products were achieved in good yields. *meta*-Substituted arylboronic acids gave analogous outcomes. Heteroaryl boronic acids also gave good yields in the optimized reaction environment.

**Table 3:** The graphite-catalyzed *ipso*-hydroxylation of arylboronic acids^a^

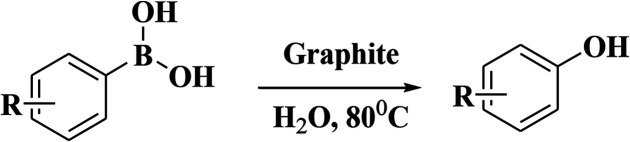
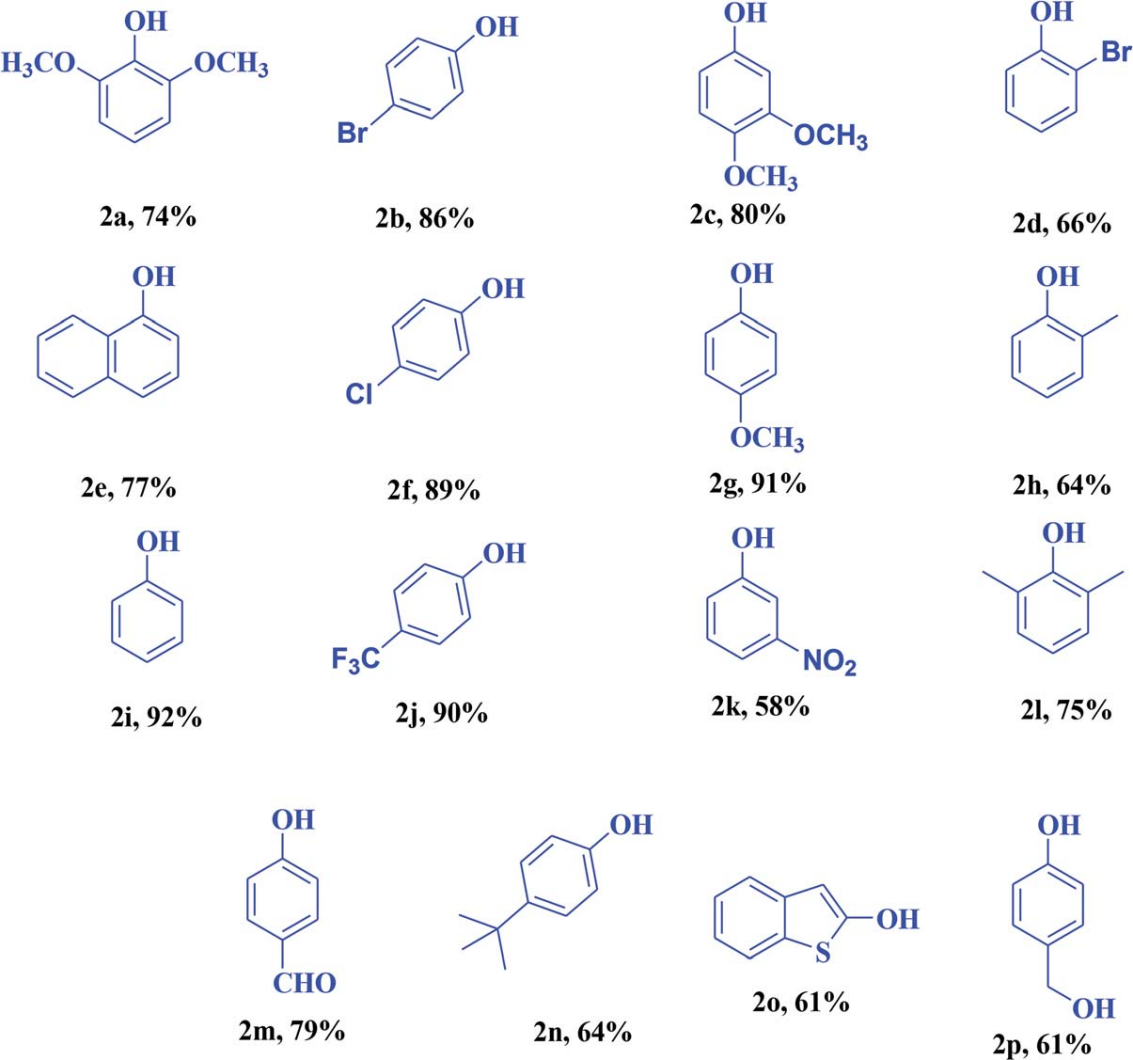

a Reactions are performed with various phenylboronic acids in water at 80 °C under air.

**Scheme 3:**
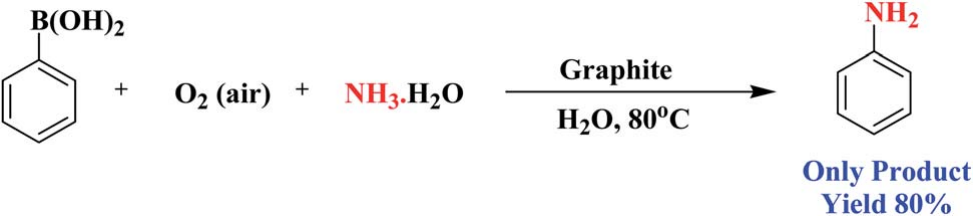
Intermolecular competition between the oxygen and nitrogen centres during the functionalization of phenylboronic acid.

Encouraged by the above results, we would like to examine whether the formation of anilines would happen when aqueous NH_3_ solution was inserted into the system due to nucleophilic competition between the nitrogen and oxygen centers. To our delight, the formation of aniline was observed instead of phenol. A probable reason for this is that the superior nucleophilic power of the nitrogen center compared to the oxygen center led to the construction of aniline instead of phenol (Scheme 3). Therefore, the reaction proceeds through the more nucleophilic center (in this case the nitrogen center) instead of the oxygen center. Similarly, when we added NaNO_2_ to the system instead of aqueous NH_3_, nitroarenes were formed.

**Scheme 4:**
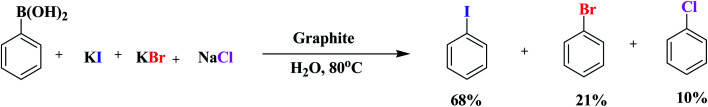
Intermolecular competition between various halide centres during the functionalization of phenylboronic acid.

Inspired by these outcomes, we used the same sustainable protocol for the iodination of arylboronic acids, using KI as an iodide source. As iodide ions have more nucleophilic power, iodobenzene was formed as the sole product. In the same way, when we used KBr in the system, bromobenzene was formed as the desired product. Unfortunately, KCl failed to give the halogenated product under these conditions. However, NaCl withstands the reaction conditions and gives the chlorinated product in good yield. Since the functionalization of phenylboronic acids at the nitrogen center was successful in the presence of an oxygen center, a competitive trial was executed to determine which phenylboronic acid functionalization would happen when three halogen reactants were added at the same time (Scheme 4). Under these conditions, iodobenzene was formed as a major product, with the order I > Br > Cl.

**Scheme 5:**
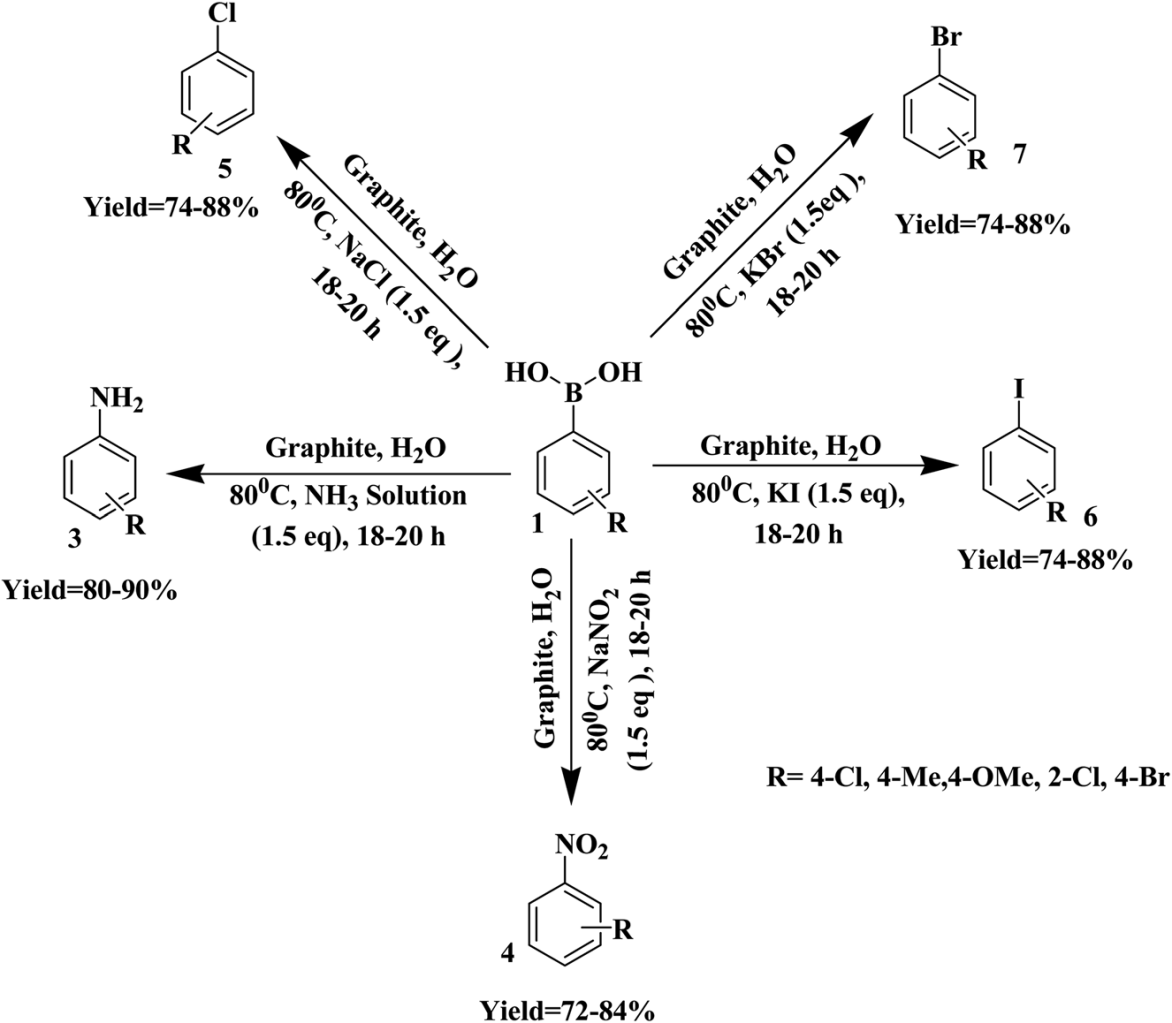
The synthesis of substituted anilines, nitroarenes, and haloarenes from arylboronic acids.

This protocol gave excellent outcomes when using NH_3_·H_2_O, NaNO_2_, KI, KBr, and NaCl as the functional-group sources. 1.5 eq. of these reagents was adequate for the reaction and the addition of additional amounts did not affect the yields remarkably. It was found that a temperature of 80 °C with a loading of 10 wt% catalyst was appropriate to give quantitative amounts of anilines, nitroarenes, and haloarenes. We employed our established conditions with the model substrates described in Scheme 5. The formation of anilines, nitroarenes, and haloarenes was not identified in the absence of atmospheric air (under a N_2_ environment), and the outcome designated that an oxidative route was involved in the synthesis of these useful compounds. To demonstrate the opportunity and boundaries of the above approach, we used a broad range of arylboronic acids. The reactions proceeded efficiently with electron-withdrawing and electron-donating arylboronic acids, but lower and more variable yields were obtained in the case of chlorobenzenes.

The heterogeneous character and excellent recyclability of graphite was confirmed (see ESI†). For recycling experiments, arylboronic acid was selected as the model substrate for all transformations. The used graphite was simply isolated *via* filtration, cleaned with ethanol, and reused for an additional four cycles. As shown in Table 4, graphite was found to be a very stable carbocatalyst for all these transformations.

**Table 4:** Recyclability experiments involving graphite for various functional group transformations^a^

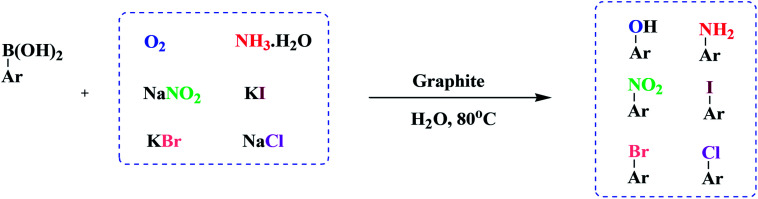				
Functional entity	First cycle [%]	Second cycle [%]	Third cycle [%]	Fourth cycle [%]
Ph-OH	92	88	86	86
Ph-NH_2_	80	78	77	77
Ph-NO_2_	72	70	68	68
Ph-I	76	72	72	71
Ph-Br	73	71	71	68
Ph-Cl	72	67	66	66

a Isolated product yield.

## 3. Experimental

### 3.1. General

See ESI.†

### 3.2. Preparation of substituted phenols (2)

A round-bottomed flask was loaded with arylboronic acid (2 mmol) and 20 wt% graphite in 20 mL of H_2_O, and the mixture was stirred at 80 °C until complete transformation was achieved (checked *via* TLC). The used graphite was removed simply *via* filtration after the transformation was complete. The filtrate was permitted to cool to atmospheric temperature and was then extracted with ethyl acetate (3 × 2 mL). The solution was then concentrated under reduced pressure and purified *via* column chromatography on silica gel (eluent, hexane : EtOAc) to furnish the pure final product.

### 3.3. Preparation of substituted anilines (3)

A round-bottomed flask was loaded with arylboronic acid (2 mmol), NH_3_ solution (1.5 eq.) and 20 wt% graphite in 20 mL of H_2_O, and the mixture was stirred at 80 °C until the complete transformation was achieved (checked *via* TLC). The used graphite catalyst was separated simply *via* filtration after the completion of the transformation. The filtrate was permitted to cool to atmospheric temperature and was then extracted with ethyl acetate (3 × 2 mL). The solution was then concentrated under reduced pressure and purified *via* column chromatography on silica gel (eluent, hexane : EtOAc) to deliver the required pure product.

### 3.4. Preparation of substituted nitroarenes (4)

A round-bottomed flask was loaded with arylboronic acid (2 mmol), NaNO_2_ (1.5 eq.) and 20 wt% graphite in 20 mL of H_2_O, and the mixture was stirred at 80 °C until the conversion was complete (monitored *via* TLC). The used graphite was separated simply *via* filtration after the completion of the transformation. The filtrate was permitted to cool to atmospheric temperature and was then extracted with ethyl acetate (3 × 2 mL). The solution was then concentrated under reduced pressure and purified *via* column chromatography on silica gel (eluent, hexane : EtOAc) to supply the final pure product.

### 3.5. Preparation of substituted haloarenes (5–7)

A round-bottomed flask was loaded with arylboronic acid (2 mmol), KCl (1.5 eq.)/KI (1.5 eq.)/KBr (1.5 eq.), and 20 wt% graphite in 20 mL of H_2_O, and the mixture was stirred at 80 °C until complete transformation was achieved (checked *via* TLC). Graphite was isolated simply *via* filtration after the transformation was complete. The filtrate was permitted to cool to atmospheric temperature and was then extracted with ethyl acetate (3 × 2 mL). The solution was then concentrated under reduced pressure and purified *via* column chromatography on silica gel (eluent, hexane : EtOAc) to offer the desired pure product.

## 4. Conclusions

A versatile and simple graphite-catalyzed process has been illustrated for the conversion of numerous functional groups on aromatic rings in an aqueous medium under air *via* the *ipso*-functionalization of phenylboronic acids. Various important aromatic compounds, *e.g.*, phenols, anilines, nitroarenes, and haloarenes, have been synthesized without using any metals, ligands, bases, or oxidants. The vital role of the aromatic π-conjugation system of graphite in assisting the reaction has been confirmed *via* various analytic techniques, *viz.*, FT-IR, Raman, XPS, SEM, and XRD analysis. *Via* control experiments, we have offered a plausible mechanistic pathway for the above transformation. The main advantage of this protocol is the use of economical and abundant graphite as an efficient carbocatalyst without any prior treatment. Overall, this strategy offers numerous advantages, such as the recyclability of the catalyst, operational simplicity, inexpensiveness, environmental friendliness, a broad substrate scope, and high yields.

## Conflicts of interest

There are no conflicts to declare.

## Supplementary Material

RA-011-D1RA01940F-s001
