# Supplementary material for: The graphite-catalyzed ipso-functionalization of arylboronic acids in an aqueous medium: metal-free access to phenols, anilines, nitroarenes, and haloarenes
Source: RSC Adv. 2021 May 19;11(29):18040–9. doi: 10.1039/d1ra01940f (PMC9033238; doi:10.1039/d1ra01940f)
Supplement: RA-011-D1RA01940F-s001 [file RA-011-D1RA01940F-s001.pdf]

Electronic Supplementary Material (ESI) for RSC Advances.
This journal is © The Royal Society of Chemistry 2021

Graphite catalyzed *ipso*-functionalization of arylboronic acids in aqueous medium: A metal-free access to phenols, anilines,nitroarenes, and haloarenes

AnshuDandia,*[a] Ruchi Sharma,[a] Pratibha Saini,[a] Ranveer Singh Badgoti,[a]Kuldeep S. Rathore,[b] Vijay Parewa,*[a]

[a]Centre of Advanced Studies, Department of Chemistry,University of Rajasthan, Jaipur, India

[b]Department of Physics, Arya College of Engineering and IT, Jaipur, India.

E-mail[a]: dranshudandia@yahoo.co.in

E-mail[a]: parewavijay.parewa@gmail.com; parewavijay@uniraj.ac.in

E-mail[b]: kuldeep_ssr@yahoo.com

Supporting Information

Contents: **Page No.**

1. General ……………………………………………………………………...... 2
2. Synthesis of substituted phenols …………………………………… 2
3. Synthesis of substituted anilines …………………………………… 3
4. Synthesis of substituted nitroarenes ……………………………………………. 3
5. Synthesis of substituted haloarenes (5,6,7) ……………………………………………. 3
6. Heterogeneous nature and recyclability of graphite ……………………………………………4
7. EDAX spectrum of fresh and reused graphite of graphite ……………………………………………5-6
8. SEM images of fresh graphite……………………………………………7
9. Solid-state 13C NMR spectra of the reused graphite.…………………………………………………8
10. EPR spectrum ………………………………………………9
11. Characterizations of the compounds ………………………………………………… 10-15
12. ^{1}H and ^{13}C-NMR spectra of the compounds ………………………………….…... 16-48

1. General

All the chemicals used were of research grade (purchased from Sigma Aldrich, Acros etc.) and used without further purification. The melting points of all compounds were determined on a Toshniwal apparatus in capillary and uncorrected. ^{1}H and ^{13}C NMR spectra were recorded in $CDCl_3$ and DMSO d_6 using TMS as an internal standard on a JEOL NMR spectrometer at 400 and 100 MHz respectively. Solid state ^{13}C NMR spectra were recorded on a JEOL NMR spectrometer (ECX400). Chemical shifts are expressed in parts per million (ppm) using tetramethylsilane (TMS) as an internal standard. The following abbreviations were used to explain the multiplicities: s = singlet, d = doublet, t = triplet and m = multiplet. Mass spectrum of representative compound was recorded on Waters-Xeevo G_2S Q-Tof. X-ray diffraction (XRD) measurements for phase determination were recorded by Philips powder diffractometer (PW3040/60) with Cu K_α radiation (1.54060nm) operating in a continuous mode to collect 2θ values with a scan rate of 0.02°/min. SEM and EDX measurements were performed using a FEI Quanta 200F SEM. The size and morphology of the synthesized material was observed by transmission electron microscopy (TEM) using a JEOL 1011 at an accelerating voltage of 200kV. (XPS) were measured on a commercial SPECS spectrometer (Germany), equipped with an Al-Kα X-ray source (1486.5eV). The UV-Vis spectra were recorded using Ocean optics USB 2000 spectrophotometer in the solution form. The Raman spectra were recorded by micro-Raman spectrometer (Jobin Yvon Horibra LABRAM-HR visible 400-1100 nm). IR spectra were recorded on a Shimadzu FT IR- 8400S spectrophotometer using KBr pellets.

2. Synthesis of substituted phenols (2)

A round-bottomed flask loaded with arylboronic acids (2mmol) and 20 wt% graphite in 20 ml H_2O were stirred at 80℃ until the complete transformation (checked by TLC). The used graphite was simply removed by filtration, after the complete transformation. Filtrate was permitted to cool to atmospheric temperature, and extracted with ethyl acetate (3X2 mL). Above solution was

concentrated under lower pressure and purified by column chromatography on silica gel (Eluent, Hexane :EtOAc(24:76)) to furnish pure final product.

3. Synthesis of substituted anilines (3)

A round-bottomed flask loaded with arylboronic acids (2mmol), NH_3 solution (1.5 eq) and 20 wt% graphite in 20 ml H_2O were stirred at 80℃ until the complete transformation (checked by TLC). The used catalyst graphite was simply separated by filtration, after completion of the transformation. Filtrate was permitted to cool to atmospheric temperature, and removed with ethyl acetate (3X2 mL). Above solution was concentrated under lower pressure and purified by column chromatography on silica gel (Eluent, Hexane :EtOAc(24:76)) to deliver pure required product.

4. Synthesis of substituted nitroarenes (4)

A round-bottomed flask loaded with arylboronic acids (2mmol), $NaNO_2$ (1.5 eq) and 20 wt% graphite in 20 ml H_2O were stirred at 80℃ until the completion of the conversion (monitored by TLC). The used graphite was simply separated by filtration, after completion of the transformation. Filtrate was permitted to cool to atmospheric temperature, and extracted with ethyl acetate (3X2 mL). Above solution was concentrated under lower pressure and purified by column chromatography on silica gel (Eluent, Hexane :EtOAc(24:76)) to supply pure final product.

5. Synthesis of substituted haloarenes (5,6,7)

A round-bottomed flask loaded with arylboronic acids (2mmol), KCl(1.5 eq)/ KI(1.5 eq)/KBr(1.5 eq) and 20 wt% graphite in 20 ml H_2O were stirred at 80℃ until the complete transformation (checked by TLC). Graphite was simply isolated by filtration, after achievement of the transformation. Filtrate was permitted to cool to atmospheric temperature, and extracted with ethyl acetate (3X2

mL). Above solution was concentrated under reduced pressure and purified by column chromatography on silica gel (Eluent, Hexane :EtOAc(24:76)) to offer pure desired product.

6. Heterogeneous nature and recyclability of graphite

To confirm the heterogeneous nature of graphite in reaction, the model reaction was carried out again under similar reaction conditions with the catalyst procured from a previous cycle. After 12 h, the catalyst was separated from the reaction mixture. The reaction was continued with filtrate for another 24h and the reaction conversion was monitored for every 1h. It was observed that further increment in conversion was not observed even after 72 h. These results revealed that reaction was occurring only due to the graphite. This whole experiment confirms the heterogeneous nature of present catalytic system.

Recycling experiments were performed by choosing the model reaction using graphite as a solid catalyst. When reaction was completed, the reaction mixture was filtered and solid precipitate was dried along with the catalyst. Then, the solid precipitate was dissolved in ethanol and catalyst was recovered by filtration. The recovered catalyst was washed with water and ethanol and reused in succeeding 4 reaction cycles with slight loss of catalytic activity. The characteristics obtained from XRD, SEM, XPS, Raman of fresh and used catalysts are similar, which suggest the retention of structure and morphology of graphite after repeated use as catalyst.

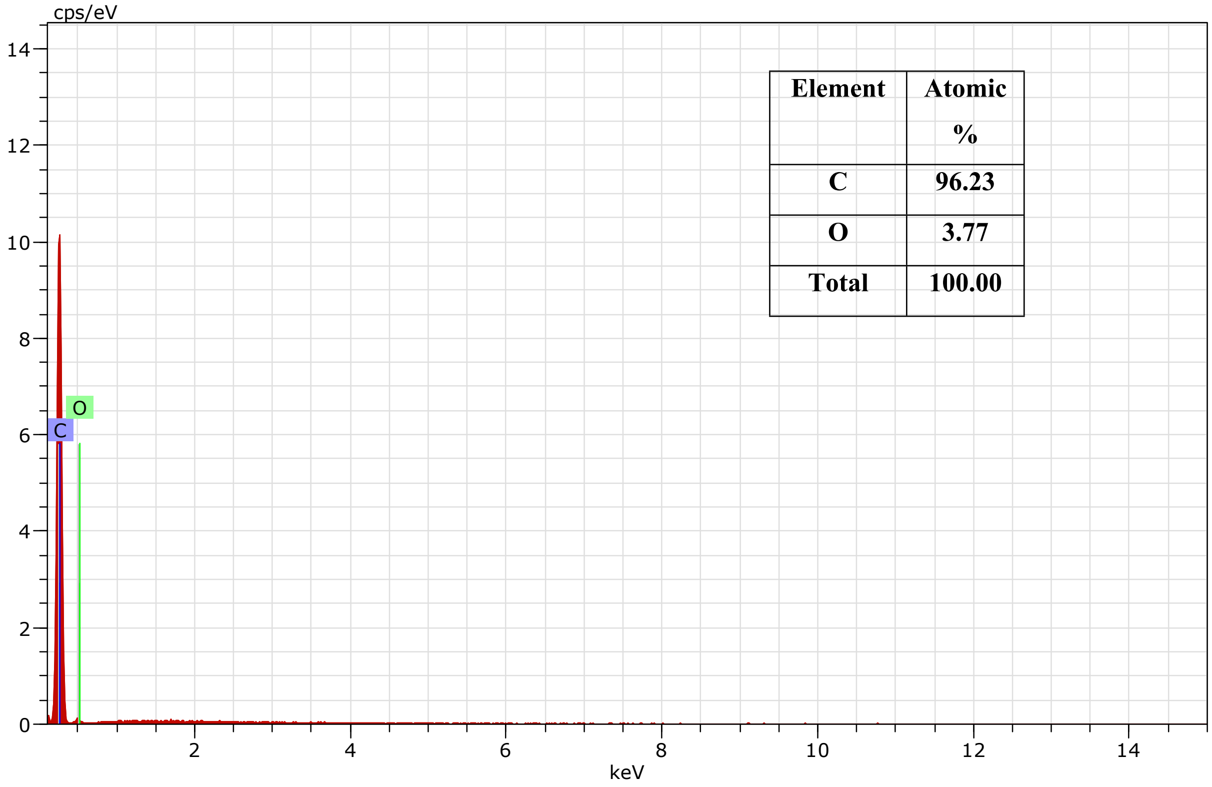

Element	Atomic %
C	96.23
O	3.77
Total	100.00

Fig.S1 EDAX spectrum of fresh graphite

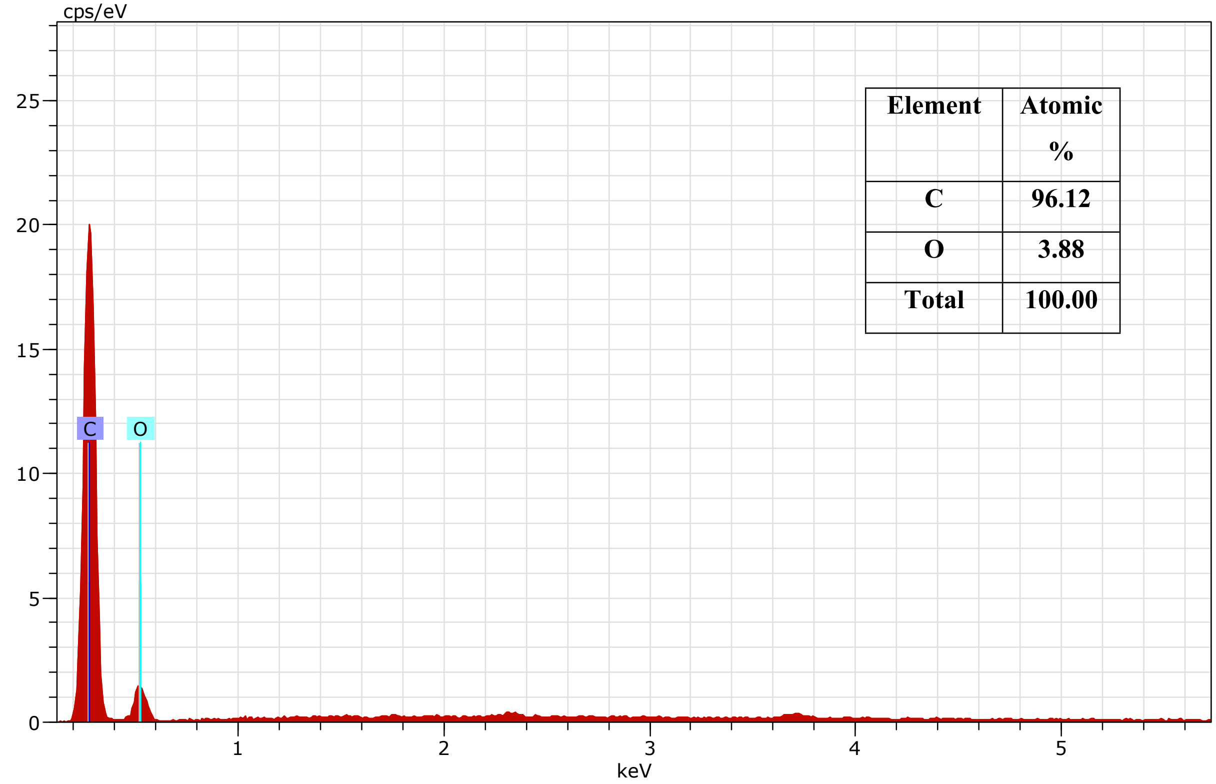

Element	Atomic %
C	96.12
O	3.88
Total	100.00

Fig.S2 EDAX spectrum of reused graphite

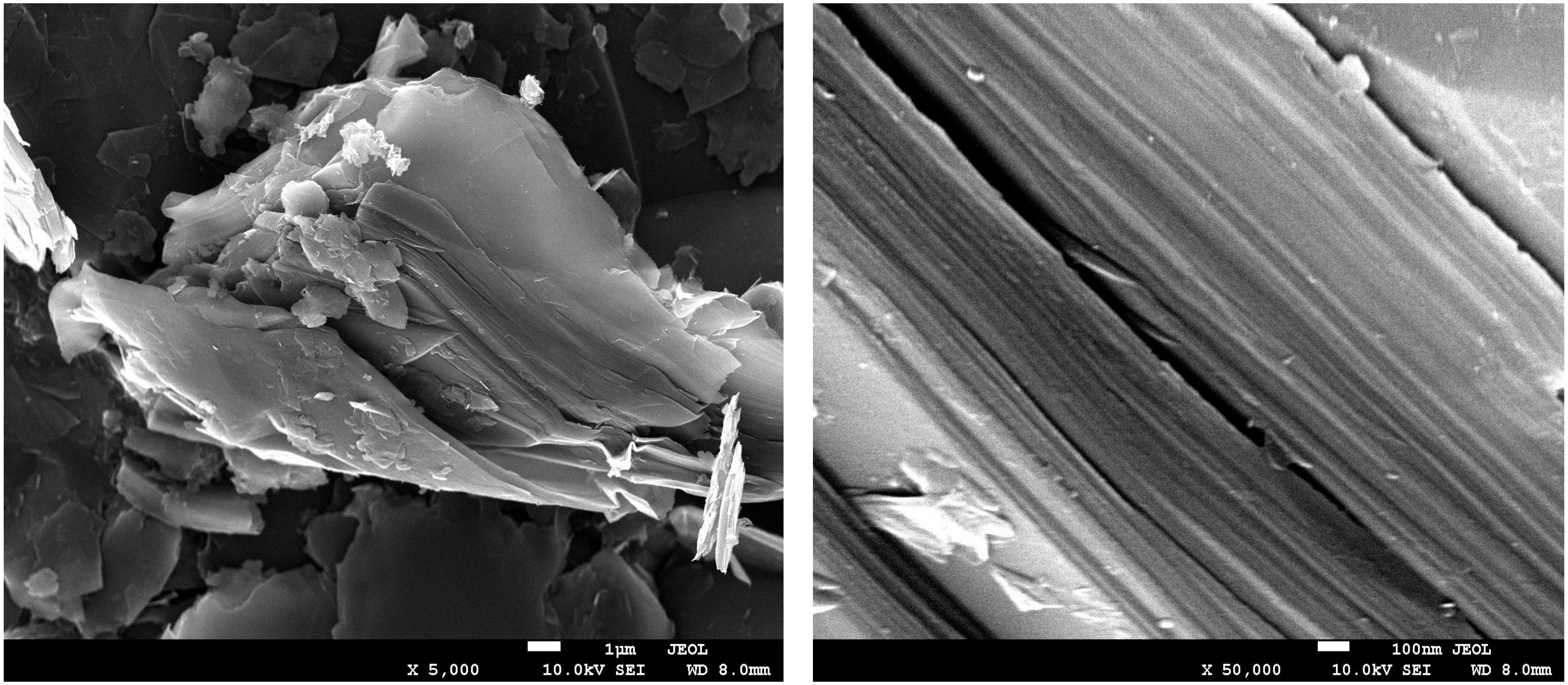

Fig.S3 SEM images of fresh graphite

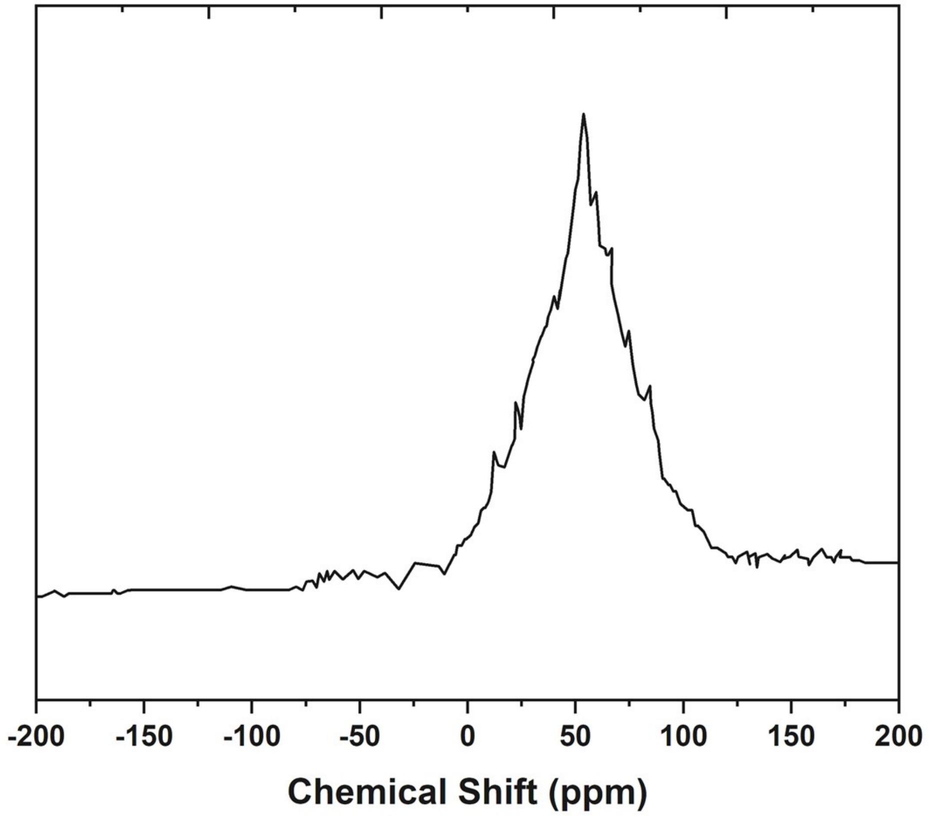

Fig.S4 Solid-state 13C NMR spectra of the reused graphite.

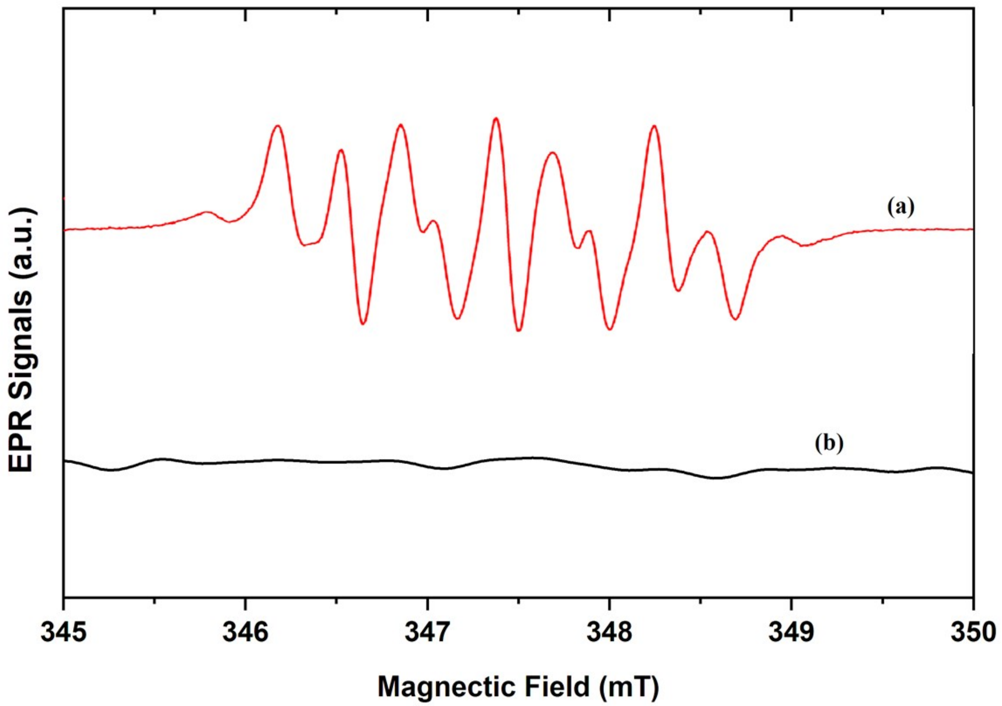

Fig.S5 EPR spectrum with DMPO (a) with catalyst (b) without catalyst

7. Spectral data of synthesized compounds (2a-7c)

2,6 di-Methoxyphenol (2a)[1]

^{1}H NMR (400 MHz, $CDCl_3$): δ ppm 7.01-6.99 (d, J=7.6, 2H, ArH), 6.80-6.76 (m, 1H, ArH), 4.65 (s, 1H, OH), 2.26 (s, 6H, OCH_3); ^{13}C NMR (100 MHz, $CDCl_3$): δ ppm 152.2, 128.7, 123.0, 120.3, 15.9; +ESI MS (m/z):155.06 $[M+H]^+$.

4-Bromophenol (2b) [4]

^{1}H NMR (400 MHz, $CDCl_3$): δ ppm 7.33-7.24 (m, 2H, ArH), 6.73-6.69 (m, 2H, ArH), 4.33 (s, 1H, OH); ^{13}C NMR (100 MHz, $CDCl_3$): δ ppm 154.7, 132.5, 117.3, 112.8; +ESI MS (m/z):172.95 $[M+H]^+$.

3,4 di-Methoxyphenol (2c)

^{1}H NMR (400 MHz, $CDCl_3$): δ ppm 8.51 (s, 1H, OH), 7.24 (s, 1H, ArH), 6.70 (s, 1H, ArH), 6.05 (s, 1H, ArH), 3.94 (s, 6H, OCH_3); ^{13}C NMR (100 MHz, $CDCl_3$): δ ppm 147.1, 143.9, 131.8, 113.0, 108.1, 107.2, 56.8, 56.1; +ESI MS (m/z):155.06 $[M+H]^+$.

2-Bromophenol (2d)[3]

^{1}H NMR (400 MHz, $CDCl_3$): δ ppm 7.11 (d, 2H, ArH), 6.92 (d, 2H, ArH), 4.21 (br s, 1H, OH); ^{13}C NMR (100 MHz, $CDCl_3$): δ ppm 140.6, 122.1, 119.7, 111.3, 106.6, 101.8; +ESI MS (m/z):172.95 $[M+H]^+$.

2-Naphtol (2e)[1]

^{1}H NMR (400 MHz, $CDCl_3$): δ ppm 7.79-7.74 (m, 2H, ArH), 7.68-7.66 (m, 1H, ArH), 7.44-7.34 (m, 2H, ArH), 7.15–7.10 (m, 2H, ArH), 5.33 (s, 1H, OH); ^{13}C NMR (100 MHz, $CDCl_3$): δ ppm 153.3, 134.6, 130.0, 129.0, 127.8, 126.6, 126.5, 123.7, 117.8, 109.6; +ESI MS (m/z): 145.06 $[M+H]^+$.

4-Chlorophenol (2f)[1]

^{1}H NMR (400 MHz, $CDCl_3$): δ ppm 7.24-7.15 (m, 2H, ArH), 6.77-6.73 (m, 2H, ArH), 4.24 (s, 1H, OH); ^{13}C NMR (100 MHz, $CDCl_3$): δ ppm 154.2, 129.6, 125.6, 116.7; +ESI MS (m/z): 129.00 $[M+H]^+$.

4-Methoxyphenol (2g)[2]

^{1}H NMR (400 MHz, $CDCl_3$): δ ppm 6.80 (s, 4H, ArH), 6.70 (br s, 1H, OH), 3.76 (s, 3H, OCH_3); ^{13}C NMR (100 MHz, $CDCl_3$): δ ppm 153.5, 149.6, 116.3, 115.2, 56.1; +ESI MS (m/z): 125.05 $[M+H]^+$.

2-Methylphenol (2h)[1]

^{1}H NMR (400 MHz, $CDCl_3$): δ ppm 7.21-6.78 (m, 2H, ArH), 6.79-6.52 (m, 2H, ArH), 4.66 (br s, 1H, OH), 1.27 (s, 3H, CH_3); ^{13}C NMR (100 MHz, $CDCl_3$): δ ppm 152.6, 136.4, 129.0, 126.7, 121.1, 112.5, 28.2; +ESI MS (m/z): 109.06 $[M+H]^+$.

Phenol (2i)[1]

^{1}H NMR (400 MHz, $CDCl_3$): δ ppm 7.36-7.31 (m, 2H, ArH), 7.08-6.98 (m, 3H, ArH), 5.12 (s, 3H, OH),; ^{13}C NMR (100 MHz, $CDCl_3$): δ ppm 155.3, 130.0, 121.3, 115.7; +ESI MS (m/z): 95.05 $[M+H]^+$.

4-(Trifluoromethyl)phenol (2j)

^{1}H NMR (400 MHz, $CDCl_3$): δ ppm 7.22 (2H, d, ArH), 6.70 (d, 2H, ArH), 5.33 (br s, 1H, OH); ^{13}C NMR (100 MHz, $CDCl_3$): δ ppm 158.5, 151.0, 140.1, 121.7, 119.2, 116.1; +ESI MS (m/z):163.03 $[M+H]^+$.

3-Nitrophenol (2k)[1]

^{1}H NMR (400 MHz, $CDCl_3$): δ ppm 7.87 (dd, 1H,ArH), 7.72–7.59 (m, 1H, ArH), 7.49 (d, 1H, ArH), 7.25 (dd, 1H, ArH), 4.93 (br s, 1H, OH); ^{13}C NMR (100 MHz, $CDCl_3$): δ ppm 160.7, 156.0, 141.9, 132.6, 126.4,111. 9; +ESI MS (m/z): 140.03 $[M+H]^+$.

2,6-Dimethylphenol (2l)[1]

^{1}H NMR (400 MHz, $CDCl_3$): δ ppm 7.61 (d, 2 H, ArH), 6.97 (d, 1H, ArH), 4.93 (s, 1H, OH), 2.65 (s, 6 H, CH_3); ^{13}C NMR (100 MHz, $CDCl_3$): δ ppm 159.5, 138.5, 126.3, 117.6, 17.3; +ESI MS (m/z):123.07 $[M+H]^+$.

4-Hydroxybenzaldehyde (2m)[1]

^{1}H NMR (400 MHz, $CDCl_3$): δ ppm 9.92 (s, 1H, CHO), 7.78 (d, 2H, ArH), 6.62 (d, 2H, ArH), 5.25 (br s, 1H, OH); ^{13}C NMR (100 MHz, $CDCl_3$): δ ppm 158.4, 138.4, 128.7, 123.9, 19.2; +ESI MS (m/z):123.04 $[M+H]^+$.

4-tert-Butylphenol (2n)[2]

^{1}H NMR (400 MHz, $CDCl_3$): δ ppm 7.67 (d, 2H, ArH), 6.65 (s, 2H, ArH), 4.89 (br s, 1H, OH), 1.29 (s, 9H, CH_3); ^{13}C NMR (100 MHz, $CDCl_3$): δ ppm 153.7, 146.9, 131.8, 128.1, 35.6, 22.0; +ESI MS (m/z):151.10 $[M+H]^+$.

Benzo[*b*]thiophen-2-ol (2o)

^{1}H NMR (400 MHz, $CDCl_3$): δ ppm 8.34 (s, 1H, OH), 8.00-7.85 (m, 2H, ArH), 7.44-7.36 (m, 2H, ArH), 4.75 (s, 1H, ArH); ^{13}C NMR (100 MHz, $CDCl_3$): δ ppm 143.4, 133.1, 125.6, 124.4, 122.6, 84.4; +ESI MS (m/z):151.10 $[M+H]^+$.

4-(Hydroxymethyl)phenol (2p)

^{1}H NMR (400 MHz, $CDCl_3$): δ ppm 7.12-7.04 (m, 2H, ArH), 6.21 (s, 2H, ArH), 5.08 (s, 2H, OH), 2.11 (s, 2H, CH_2); ^{13}C NMR (100 MHz, $CDCl_3$): δ ppm 151.5, 141.7, 134.8, 126.0, 69.6; +ESI MS (m/z):151.01 $[M+H]^+$.

Aniline (3a)[2]

^{1}H NMR (400 MHz, $CDCl_3$):δ ppm 7.23-7.19 (m, 2H, ArH), 6.83-6.81 (d, J=7.2, 1H, ArH), 6.72-6.70 (m, 2H, ArH), 3.62 (br s, 2H, NH_2); ^{13}C NMR (100 MHz, $CDCl_3$): δ ppm 146.5, 129.4, 118.6, 115.2; +ESI MS (m/z):94.12 $[M+H]^+$.

4-Chloroaniline (3b)[2]

^{1}H NMR (400 MHz, $CDCl_3$):δ ppm 7.21 (m, 2H, ArH), 6.98 (m, 2H, ArH), 3.27 (s, 2H, NH_2); ^{13}C NMR (100 MHz, $CDCl_3$): δ ppm 155.8, 136.7, 127.9, 119.0; +ESI MS (m/z):128.02 $[M+H]^+$.

4-Methoxyaniline (3c)[2]

^{1}H NMR δ (400 MHz, $CDCl_3$): 6.75-6.72 (m, 2H, ArH), 6.65-6.62 (m, 2H, ArH), 3.73 (s, 3H, OCH_3), 3.41 (s, 2H, NH_2); ^{13}C NMR (100 MHz, $CDCl_3$): δ ppm 152.8, 140.0, 116.5, 114.8, 55.8; +ESI MS (m/z): 124.07 $[M+H]^+$.

Nitrobenzene (4a)[5]

^1H NMR (400 MHz, $CDCl_3$): δ ppm 8.18-8.15 (m, 2H, ArH), 7.68-7.64 (m, 1H, ArH), 7.52-7.48 (m, 2H, ArH); ^{13}C NMR (100 MHz, $CDCl_3$): δ ppm 148.2, 134.7, 129.3, 123.4; +ESI MS (m/z):124.10 $[M+H]^+$.

1-Methyl-4-nitro-benzene (4b)[5]

^1H NMR (400 MHz, $CDCl_3$): δ ppm 7.91 (d, 2H, ArH), 7.23 (d, 2H, ArH), 2.9 (s, 3H, CH_3); ^{13}C NMR (100 MHz, $CDCl_3$): δ ppm 151.7, 143.0, 134.9, 128.9, 23.9; +ESI MS (m/z):138.05 $[M+H]^+$.

1-Methyl-3-nitro-benzene (4c)

^1H NMR (400 MHz, $CDCl_3$): δ ppm 7.92-7.90 (m, 2H, ArH), 7.43-7.41 (m, 1H, ArH), 7.35-7.31 (m, 1H, ArH), 2.37 (s, 3H, CH_3); ^{13}C NMR (100 MHz, $CDCl_3$): δ ppm 148.1, 139.8, 135.4, 129.1, 123.7, 120.6, 21.1; +ESI MS (m/z):138.13 $[M+H]^+$.

1-Bromo-4-nitrobenzene (4d)[5]

^1H NMR (400 MHz, $CDCl_3$): δ ppm 7.94 (dd, 2H, ArH), 7.67 (dd, 2H, ArH); ^{13}C NMR (100 MHz, $CDCl_3$): δ ppm 141.7, 137.9, 126.9, 119.3; +ESI MS (m/z):201.94 $[M+H]^+$.

4-Methoxychlorobenzene (5a)

^1H NMR (400 MHz, $CDCl_3$): δ ppm 7.94-6.12 (m, 4H, ArH), 3.94 (s, 3H, OCH_3); ^{13}C NMR (100 MHz, $CDCl_3$): δ ppm 154.8, 145.7, 137.2, 122.7, 108.5, 56.8; +ESI MS (m/z):143.02 $[M+H]^+$.

4-Methylchlorobenzene (5b)

^1H NMR (400 MHz, $CDCl_3$): δ ppm 7.12-6.45 (m, 4H, ArH), 2.94 (s, 3H, CH_3); ^{13}C NMR (100 MHz, $CDCl_3$): δ ppm 151.9. 143.3, 132.4, 124.8, 109.3, 20.2; +ESI MS (m/z):127.02 $[M+H]^+$.

1-Chloro-3-methyl-benzene (5c)

^1H NMR (400 MHz, $CDCl_3$): δ ppm 7.22-7.16 (m, 3H, ArH), 7.08-7.06 (m, 1H, ArH), 2.36 (s, 3H, CH_3); ^{13}C NMR (100 MHz, $CDCl_3$): δ ppm 139.9, 134.1, 129.5, 129.2, 127.3, 125.6, 21.2; +ESI MS (m/z):127.02 $[M+H]^+$.

4-Methoxyiodobenzene (6a)[6]

^{1}H NMR (400 MHz, $CDCl_3$): δ ppm 6.94-6.17 (m, 4H, ArH), 3.84 (s, 3H, OCH_3); ^{13}C NMR (100 MHz, $CDCl_3$): δ ppm 159.7. 141.9, 136.9, 121.3, 103.7, 55.2; +ESI MS (m/z):234.95 $[M+H]^+$.

4-Methyliodobenzene (6b)[7]

^{1}H NMR (400 MHz, $CDCl_3$): δ ppm 7.60-7.58 (m, 2H, ArH), 6.95-6.93 (d, J=8, 2H, ArH), 2.32 (s, 3H, CH_3); ^{13}C NMR (100 MHz, $CDCl_3$): δ ppm 137.5, 137.3, 131.3, 90.4, 21.2; +ESI MS (m/z):218.96 $[M+H]^+$.

4-Methoxybromobenzene (7a)[7]

^{1}H NMR (400 MHz, $CDCl_3$): δ ppm 7.04-6.34 (m, 4H, ArH), 3.56 (s, 3H, OCH_3); ^{13}C NMR (100 MHz, $CDCl_3$): δ ppm 151.5, 146.4, 137.3, 122.5, 113.5, 54.9; +ESI MS (m/z):186.97 $[M+H]^+$.

4-Methylbromobenzene (7b)[7]

^{1}H NMR (400 MHz, $CDCl_3$): δ ppm 7.38-7.36 (m, 2H, ArH), 7.06-7.04 (d, J=8, 2H, ArH), 2.31 (s, 3H, CH_3); ^{13}C NMR (100 MHz, $CDCl_3$): δ ppm 136.8, 131.3, 130.9, 119.1, 21.0; +ESI MS (m/z):170.97 $[M+H]^+$.

Bromobenzene (7c)[7]

^{1}H NMR (400 MHz, $CDCl_3$): δ ppm 7.56-7.54 (m, 2H, ArH), 7.33-7.31 (m, 1H, ArH), 7.29-7.25 (m, 2H, ArH),; ^{13}C NMR (100 MHz, $CDCl_3$): δ ppm 131.3, 129.8, 126.7, 122.3; +ESI MS (m/z):156.95 $[M+H]^+$.

References

1. Kotoučová, H., Strnadová, I., Kovandová, M., Chudoba, J., Dvořáková, H., & Cibulka, R. (2014). *Organic & biomolecular chemistry*, *12*(13), 2137-2142.

2. Qi, H. L., Chen, D. S., Ye, J. S., & Huang, J. M. (2013). *The Journal of organic chemistry*, *78*(15), 7482-7487.

3. Sideri, I. K., Voutyritsa, E., & Kokotos, C. G. (2018). *Synlett*, *14*(10), 1324-1328.

4. Matsui, K., Ishigami, T., Yamaguchi, T., Yamaguchi, E., Tada, N., Miura, T., & Itoh, A. (2014). *Synlett*, *25*(18), 2613-2616.

5. Zarei, M., Noroozizadeh, E., Moosavi-Zare, A. R., & Zolfigol, M. A. (2018). *The Journal of organic chemistry*, *83*(7), 3645-3650.

6. Leas, D. A., Dong, Y., Vennerstrom, J. L., & Stack, D. E. (2017). *Organic letters*, *19*(10), 2518-2521.

7. Leas, D. A., Dong, Y., Vennerstrom, J. L., & Stack, D. E. (2017). *Organic letters*, *19*(10), 2518-2521.

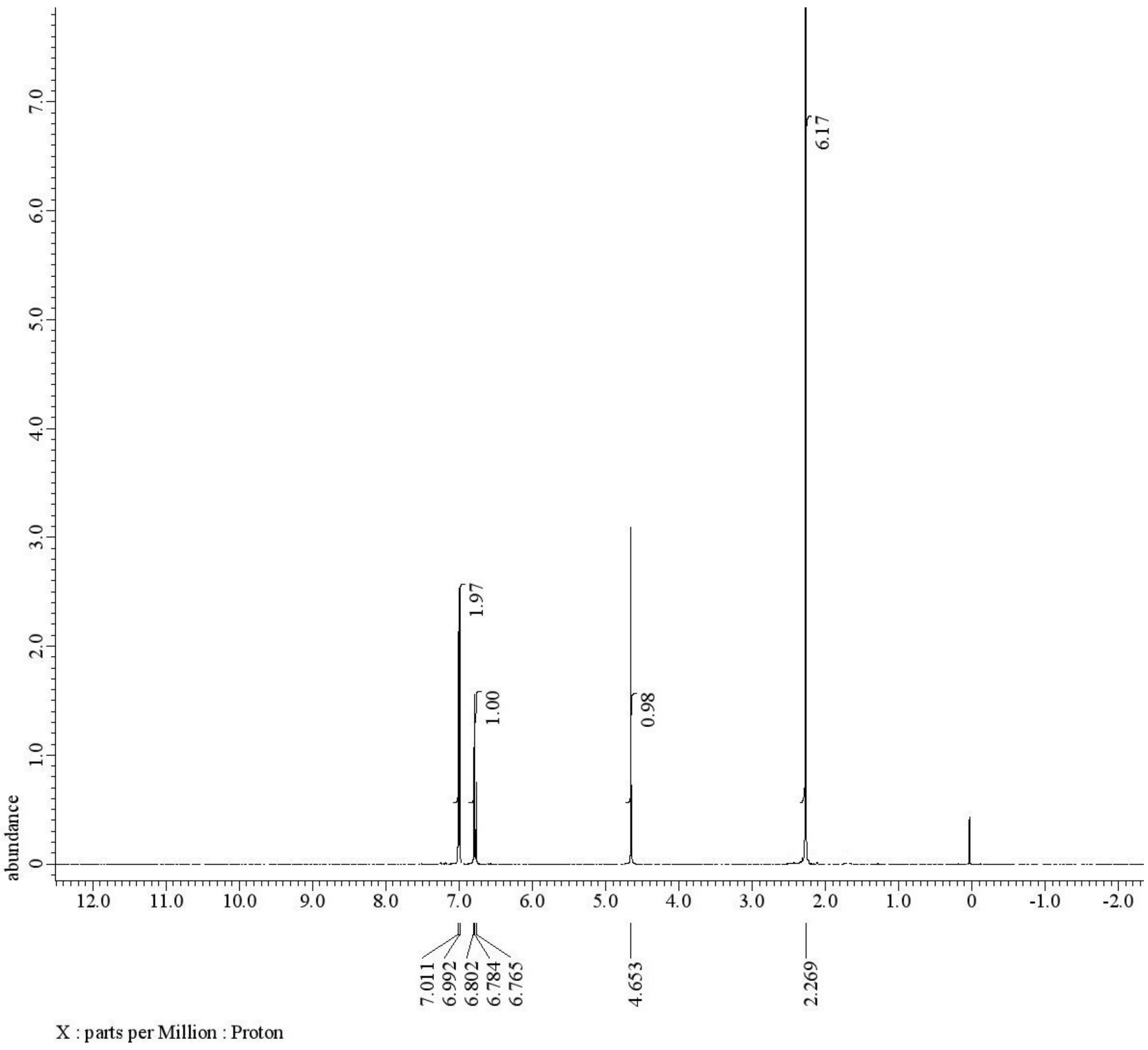

Figure 1. ^{1}H NMR spectrum of (**2a**)

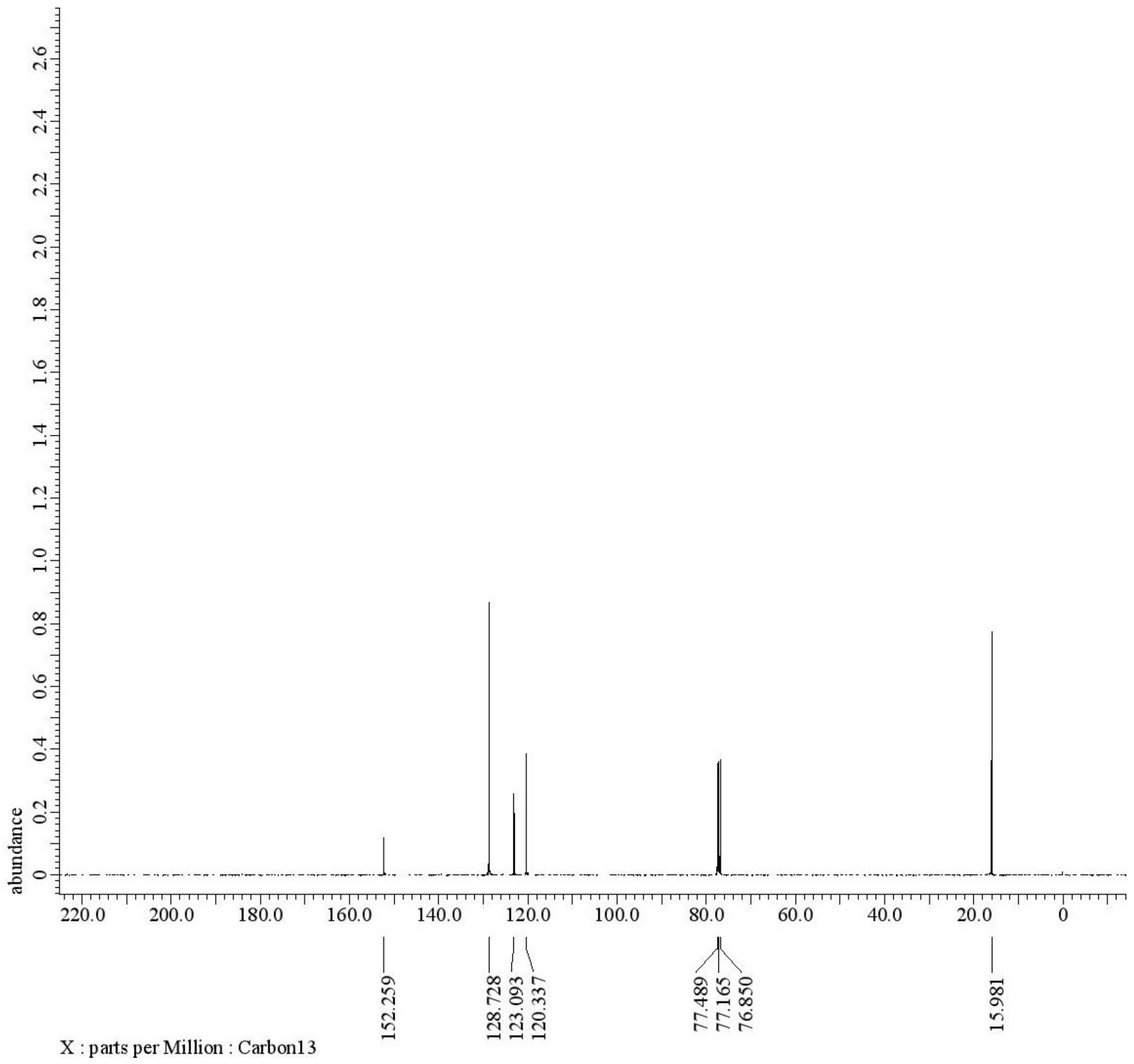

Figure 2. ^{13}C NMR spectrum of (**2a**)

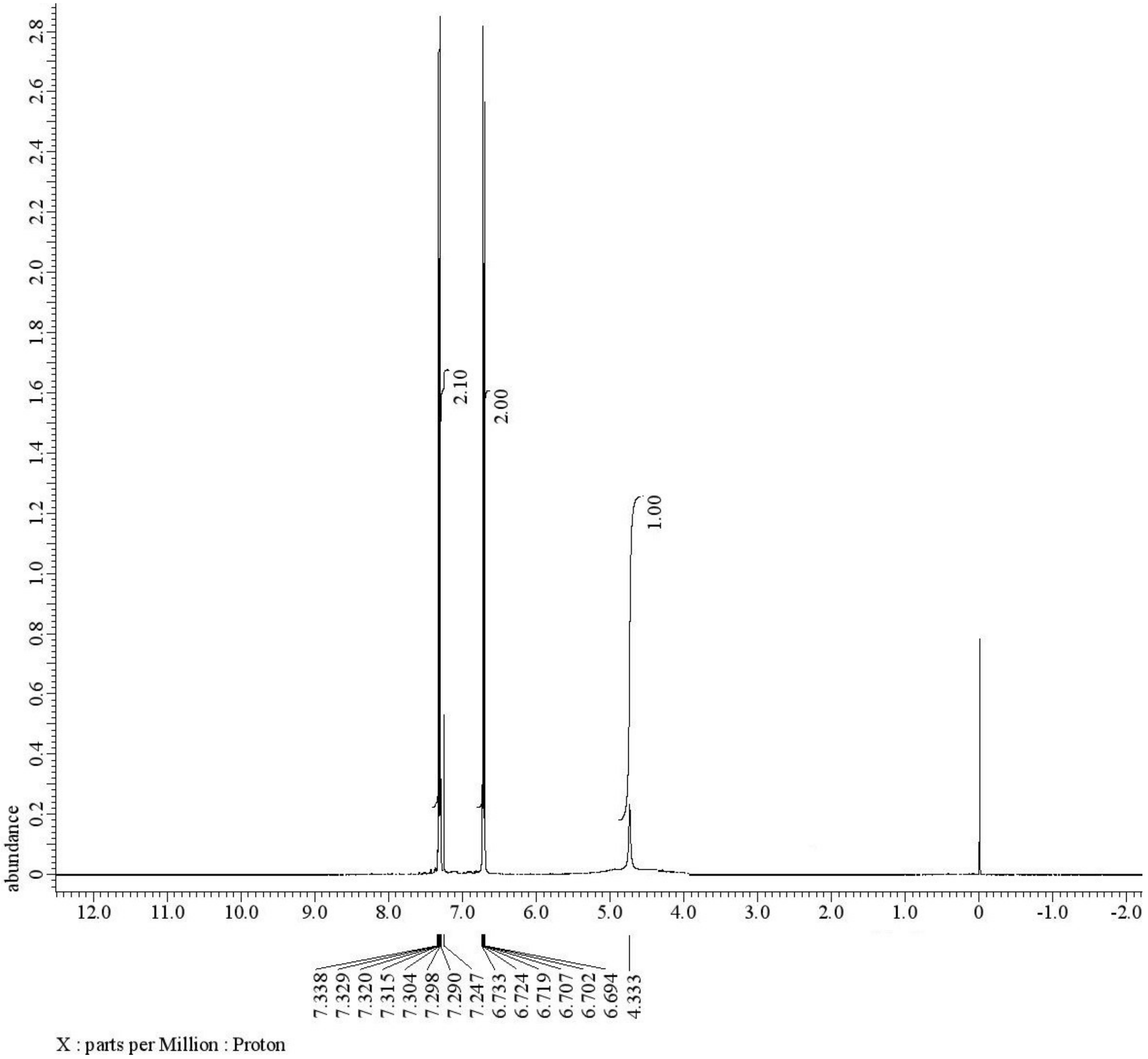

Figure 3. ^{1}H NMR spectrum of (**2b**)

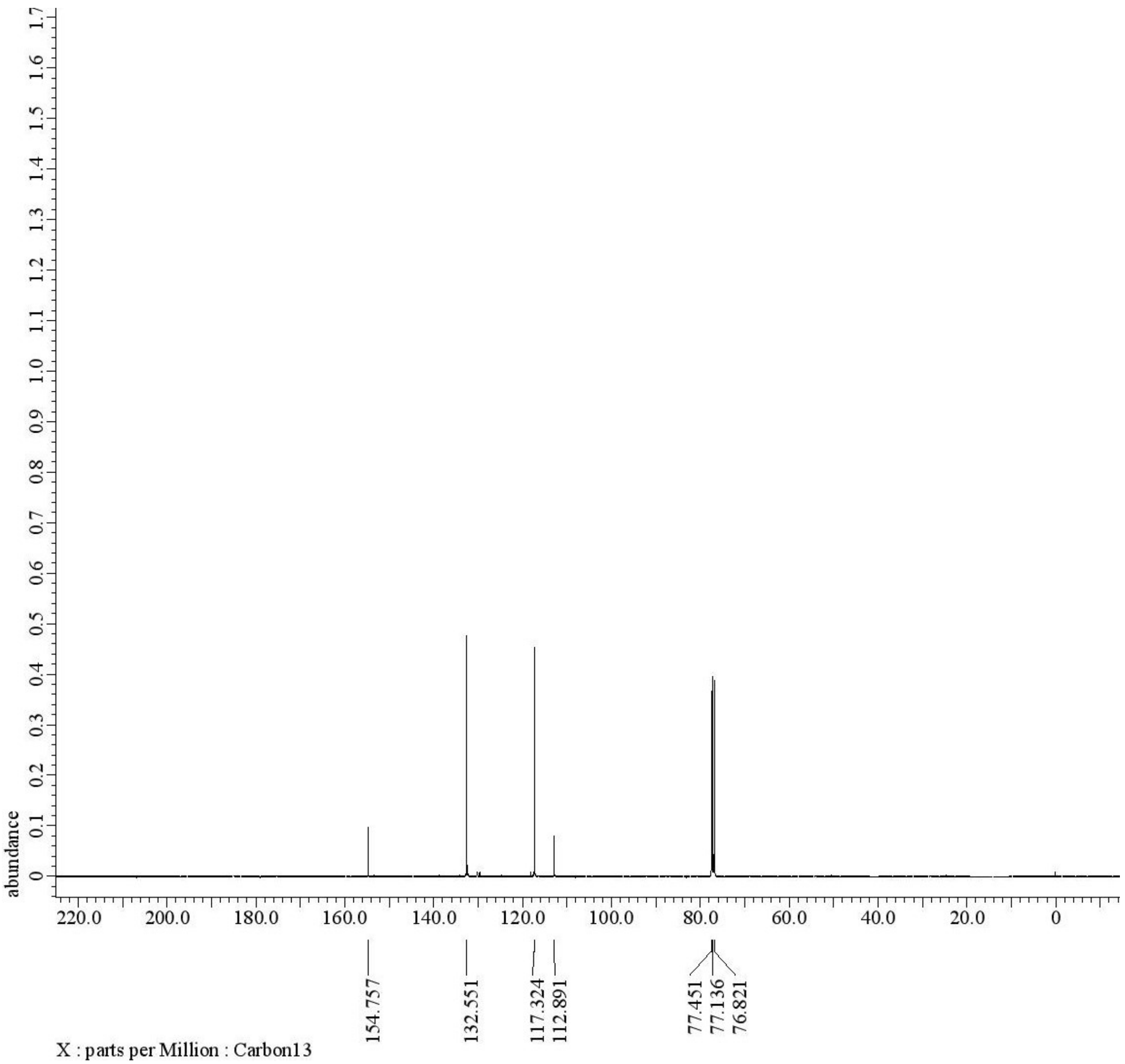

Figure 4. ^{13}C NMR spectrum of (**2b**)

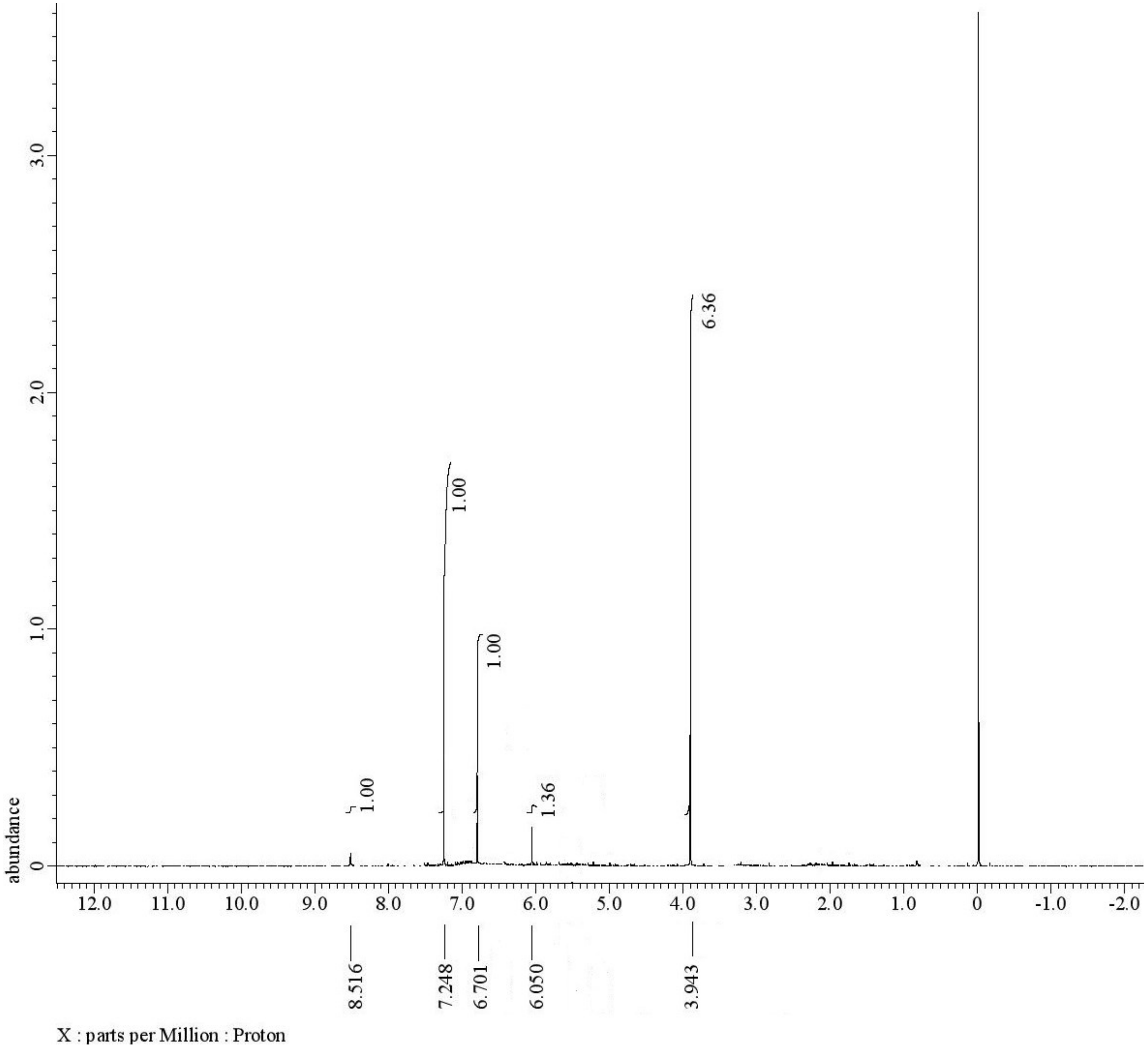

Figure 5. ^{1}H NMR spectrum of (**2c**)

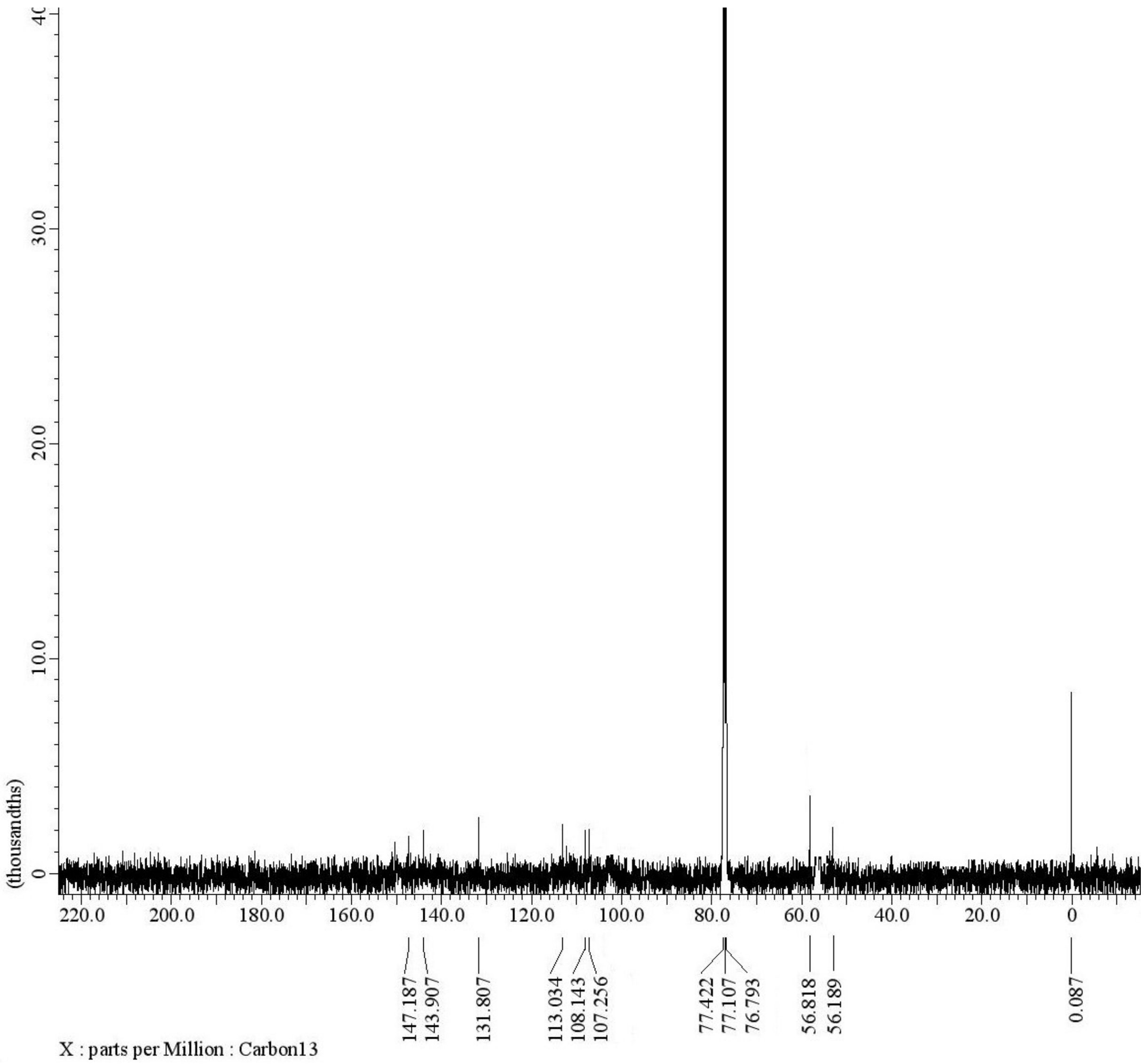

Figure 6. ^{13}C NMR spectrum of (**2c**)

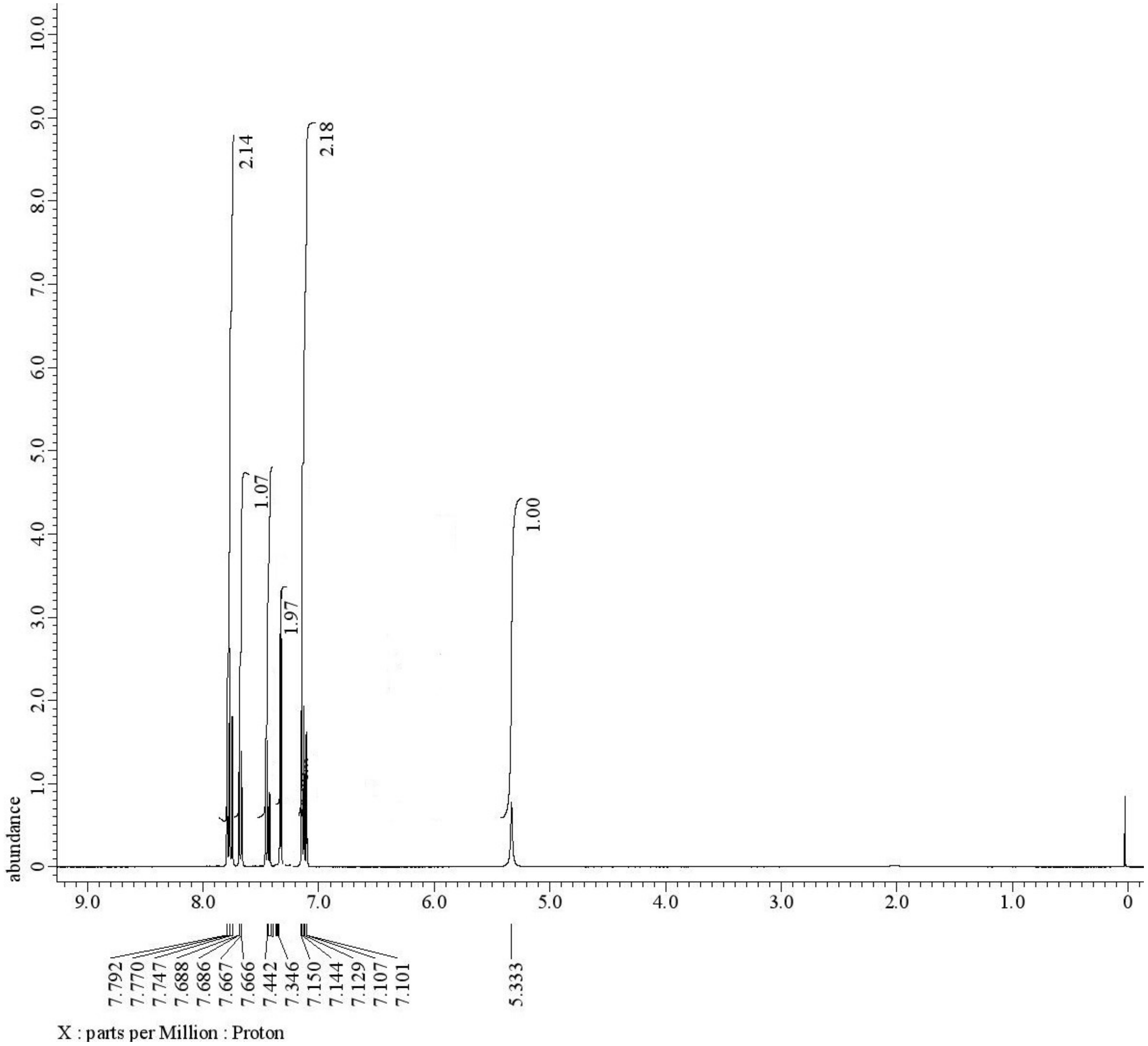

Figure 7. ^{1}H NMR spectrum of (**2e**)

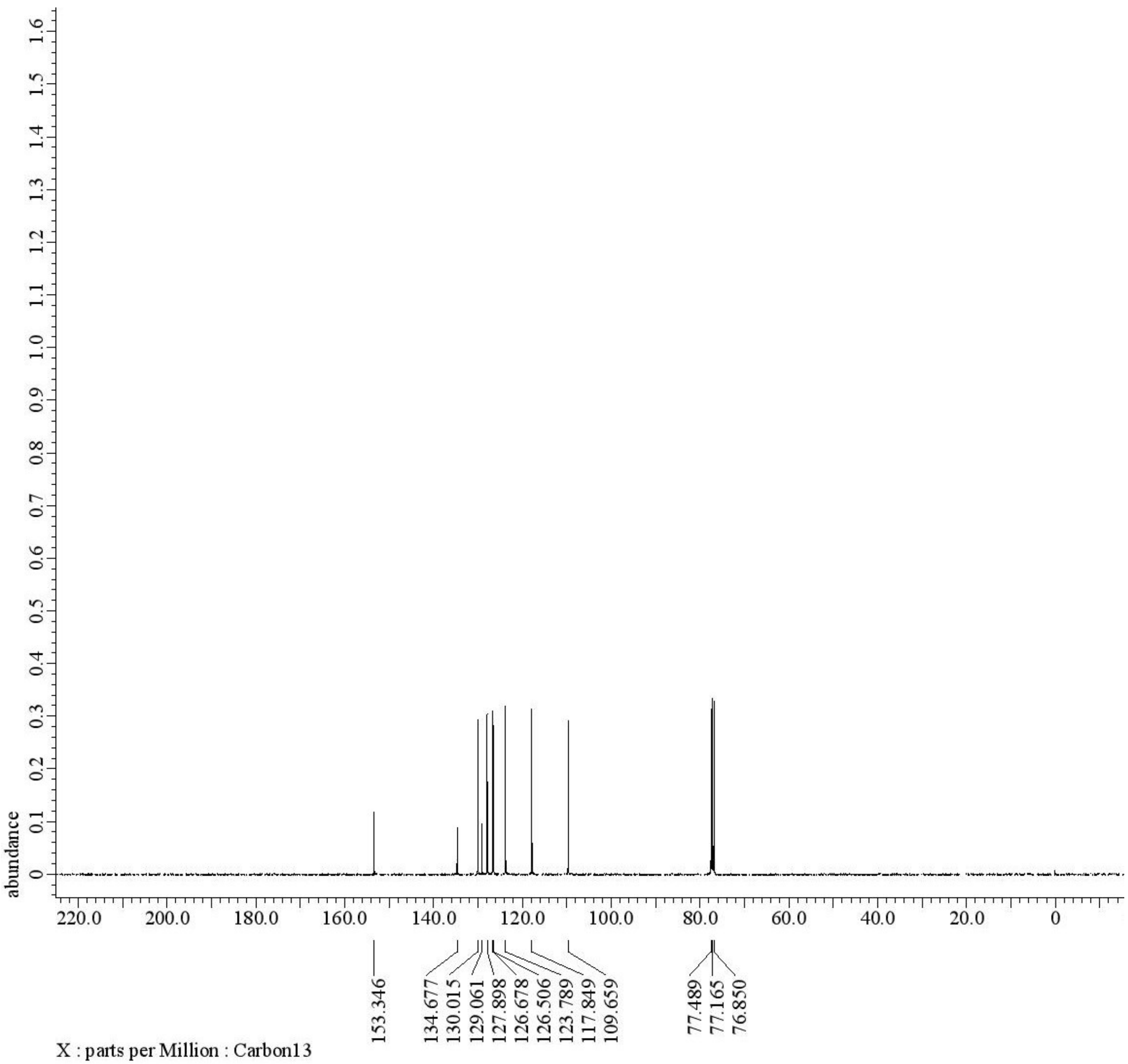

Figure 8. ^{13}C NMR spectrum of (**2e**)

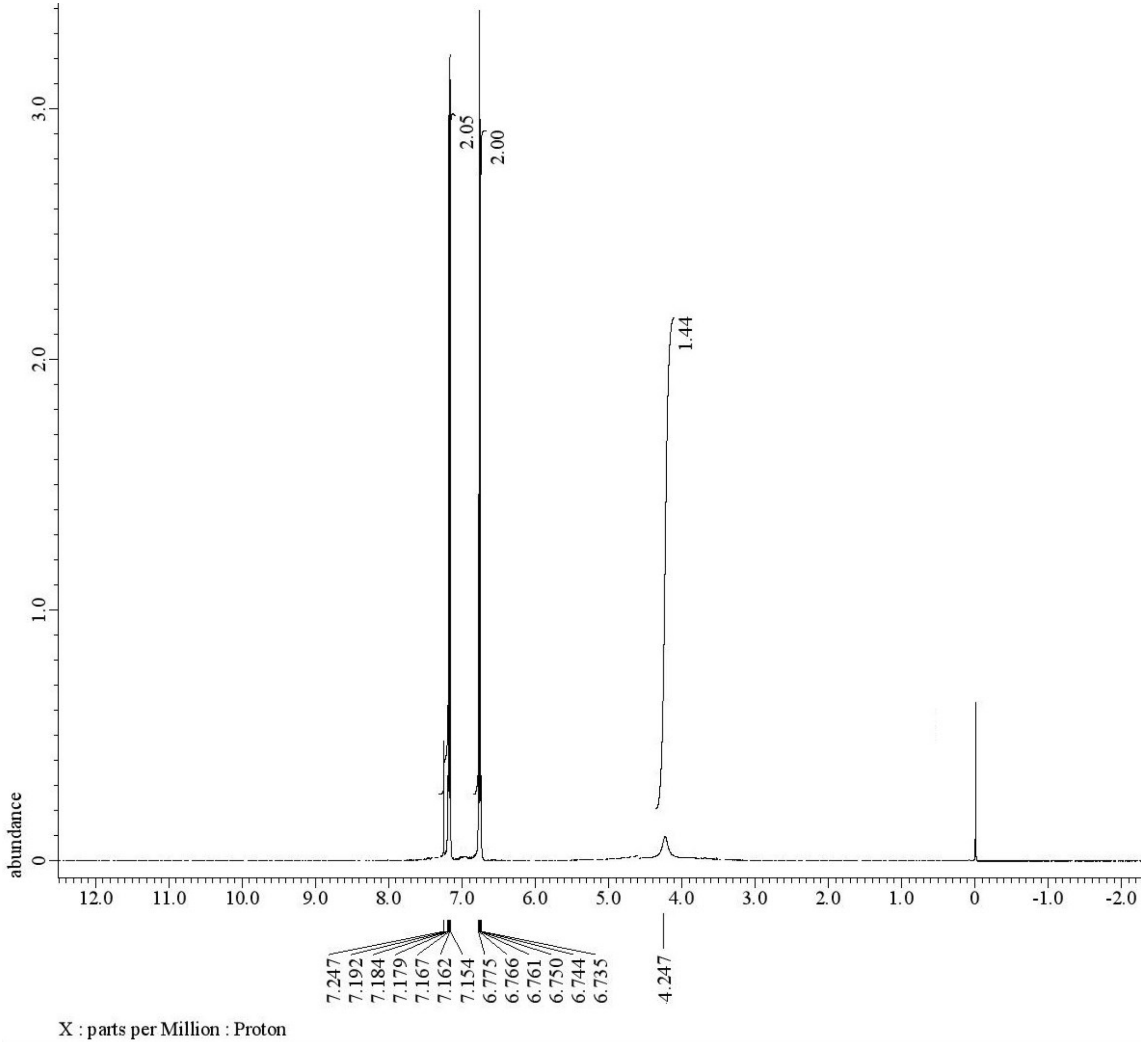

Figure 9. ^{1}H NMR spectrum of (**2f**)

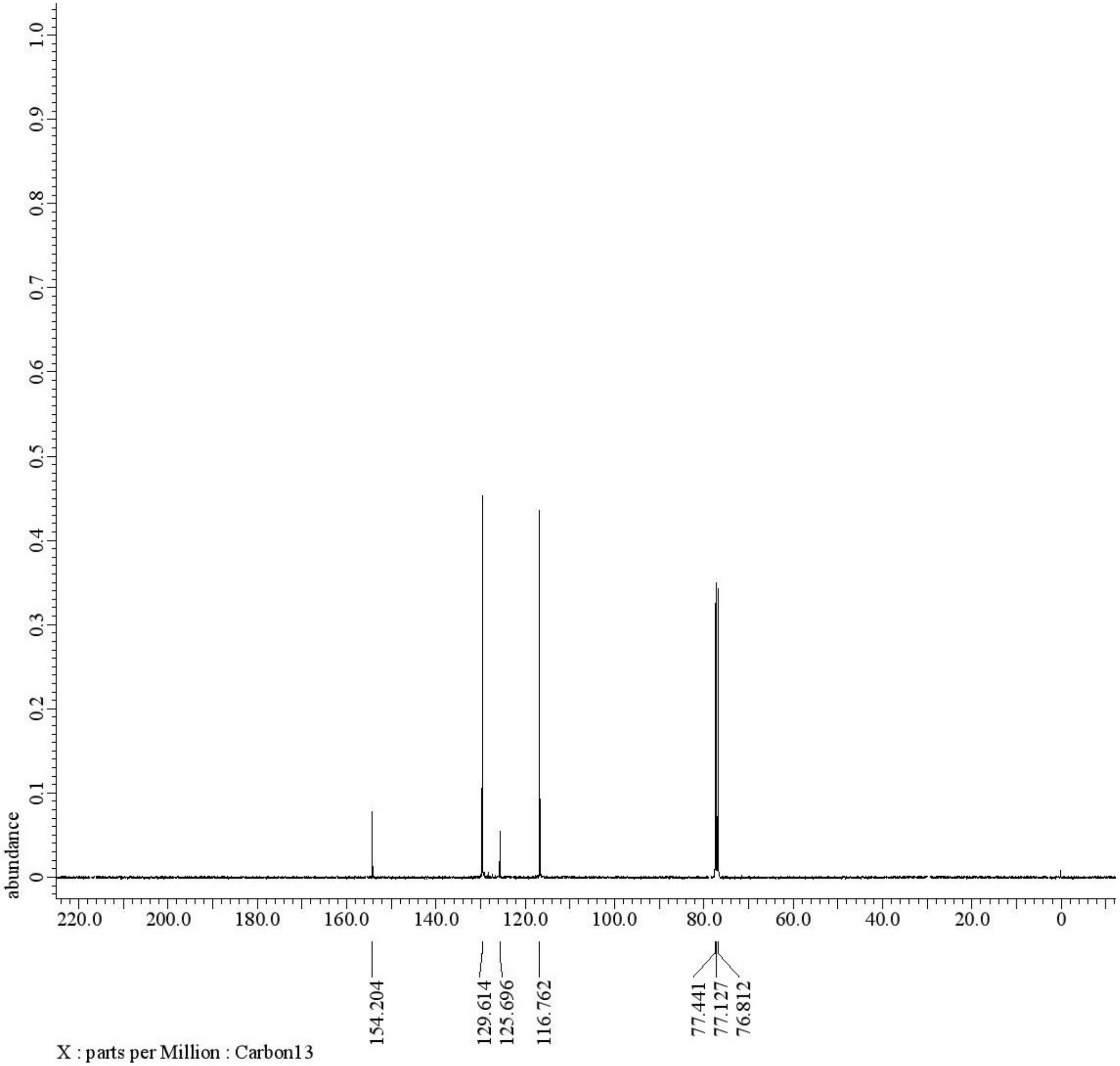

Figure 10. ^{13}C NMR spectrum of (**2f**)

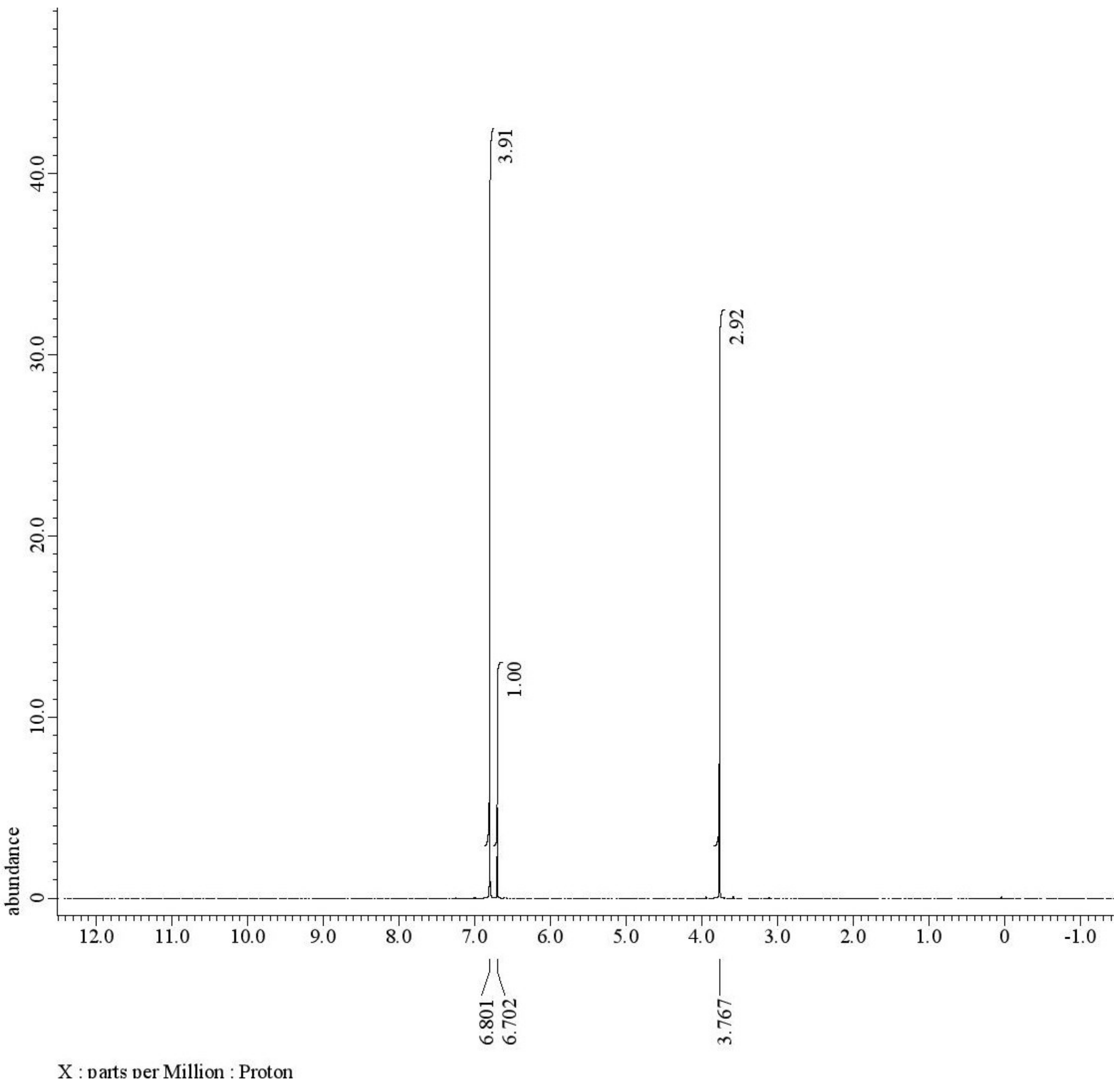

Figure 11. ^{1}H NMR spectrum of (**2g**)

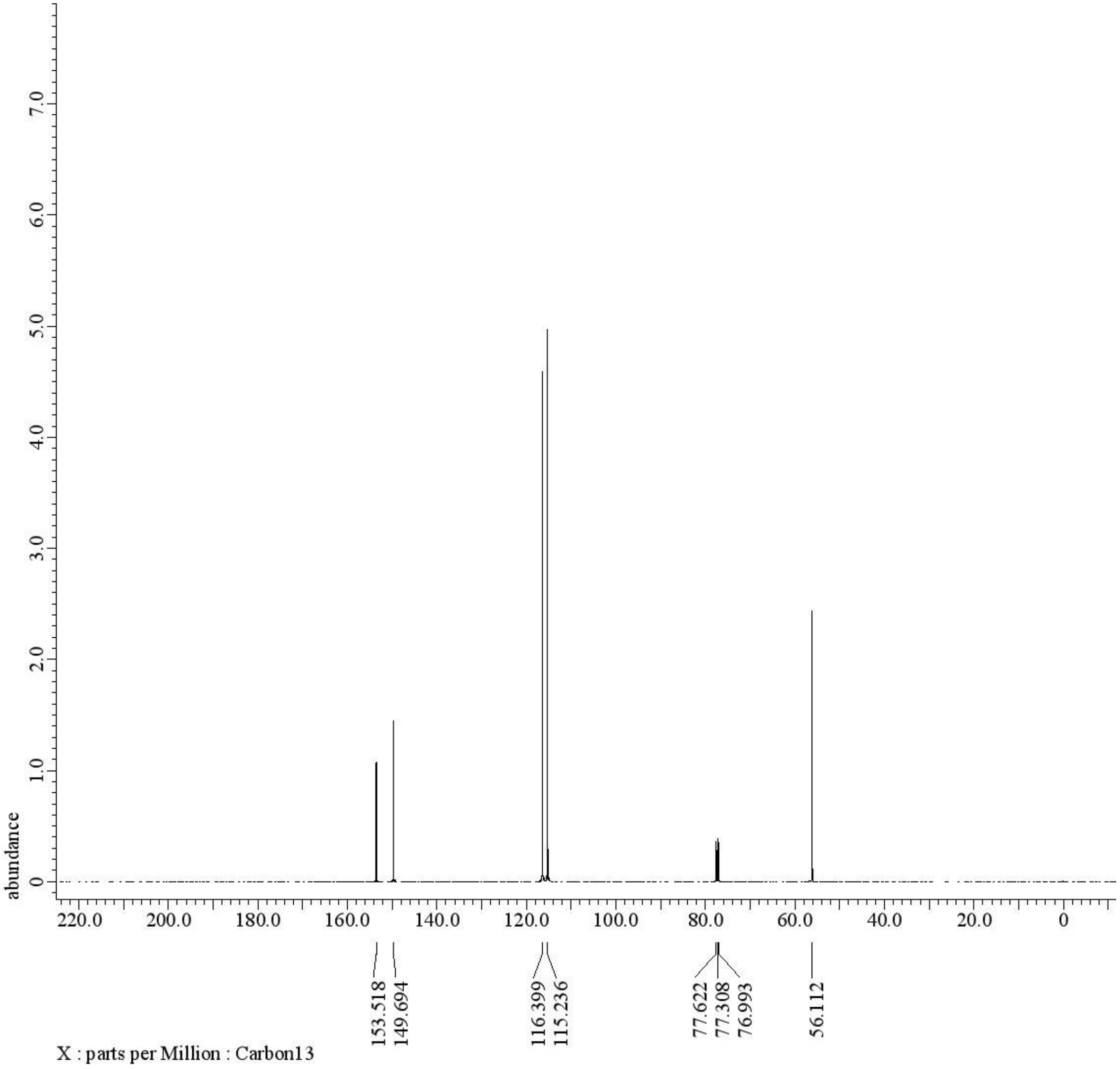

Figure 12. ^{13}C NMR spectrum of (**2g**)

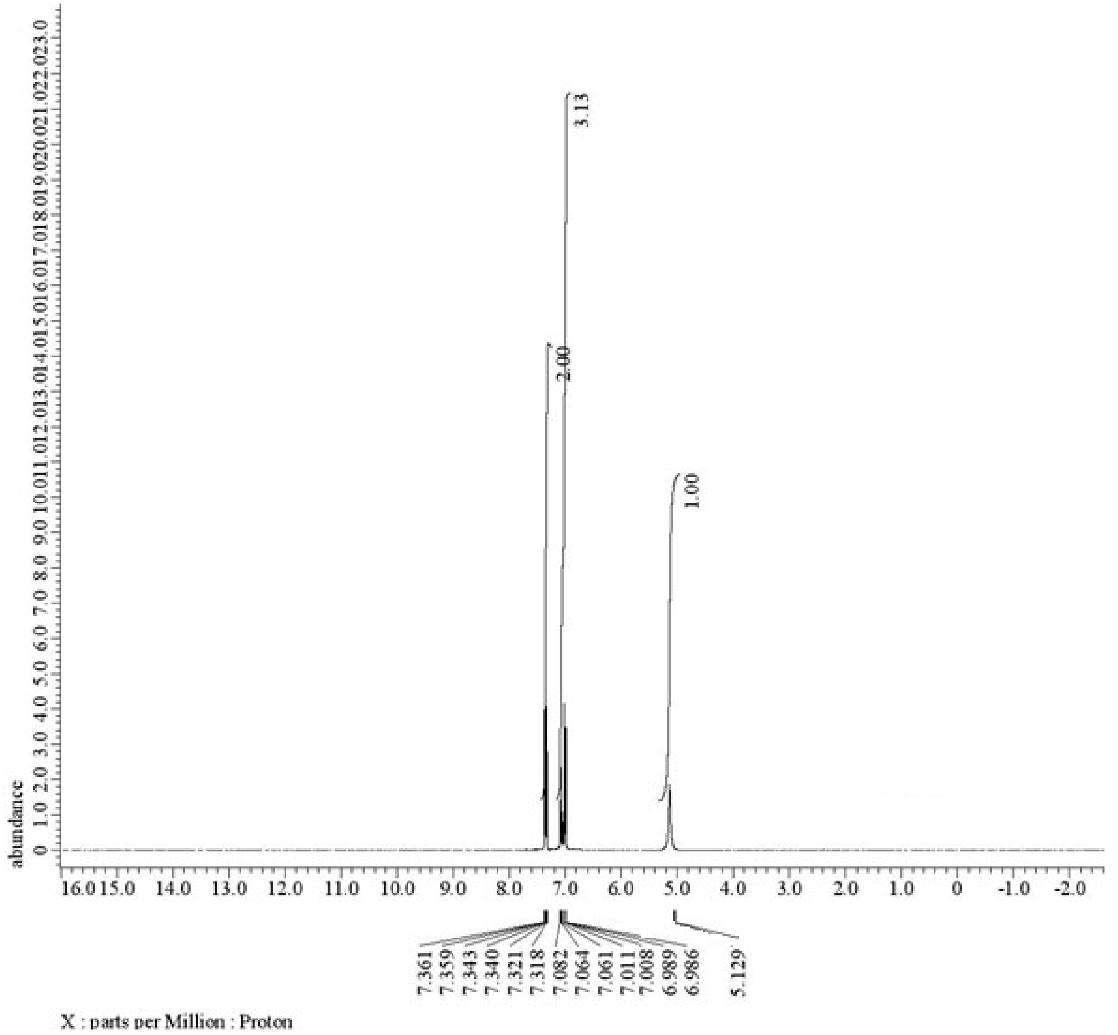

Figure 13. ^{1}H NMR spectrum of (**2i**)

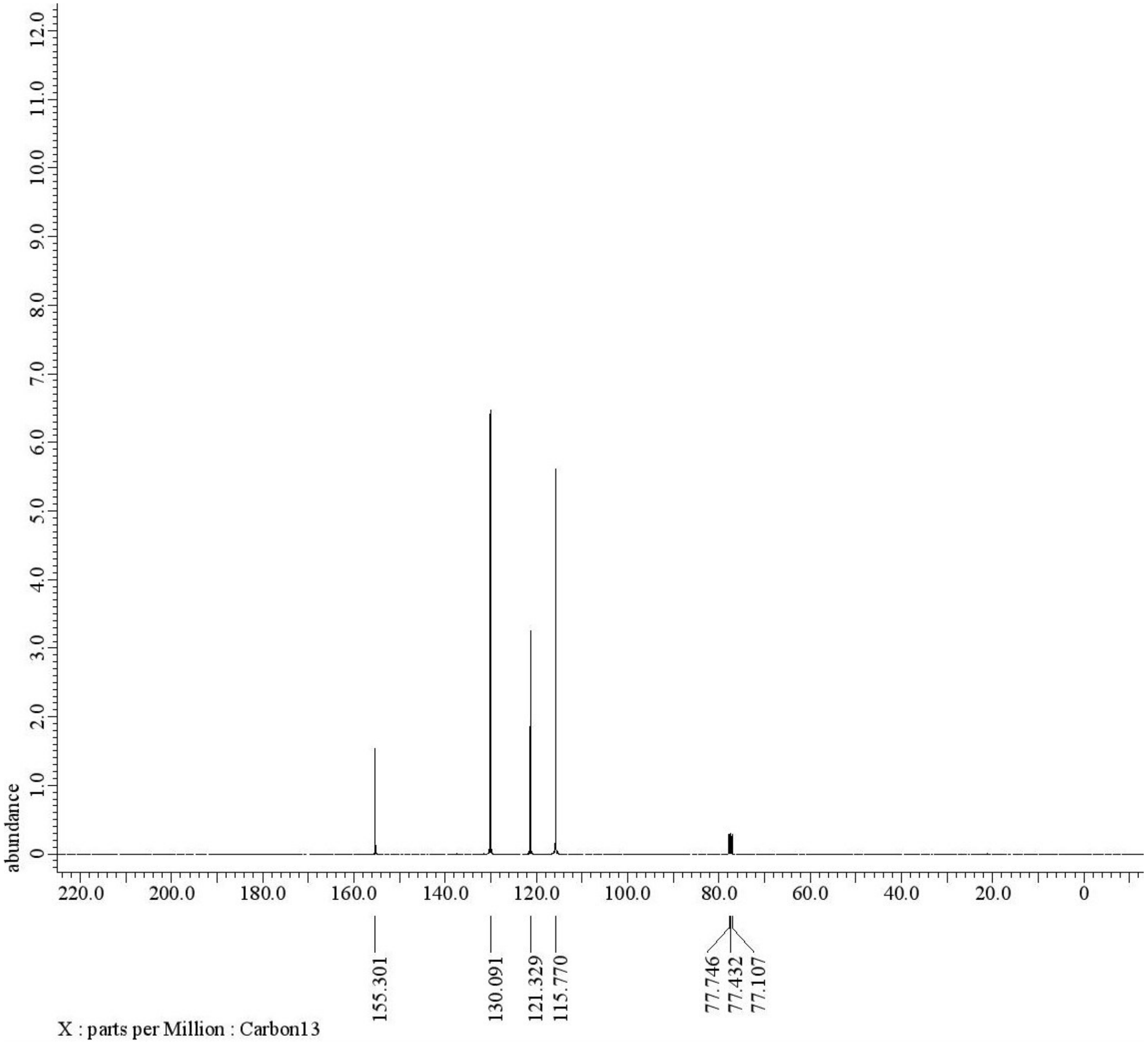

Figure 14. ^{13}C NMR spectrum of (**2i**)

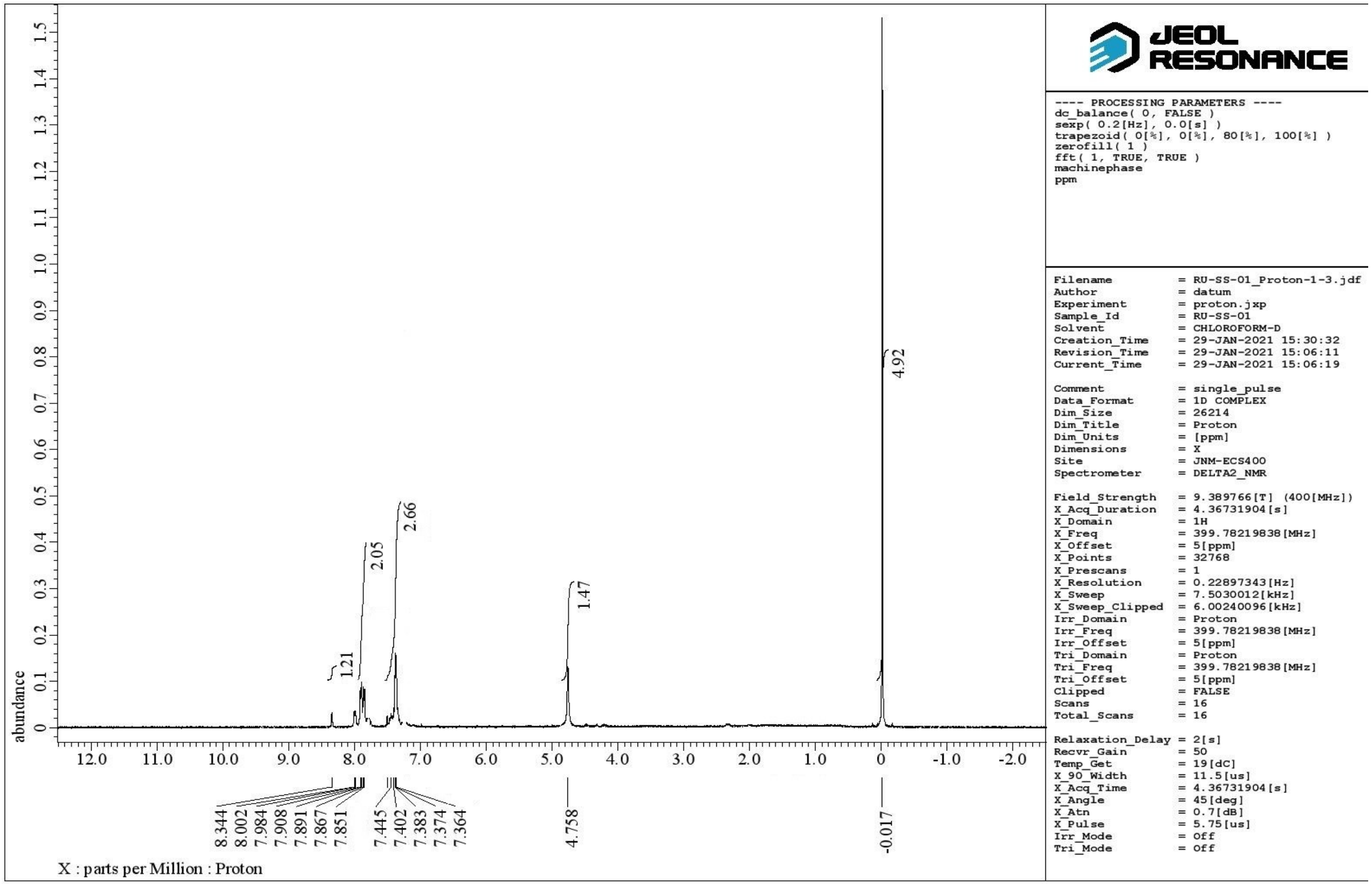

Figure 15. ^{1}H NMR spectrum of (**2o**)

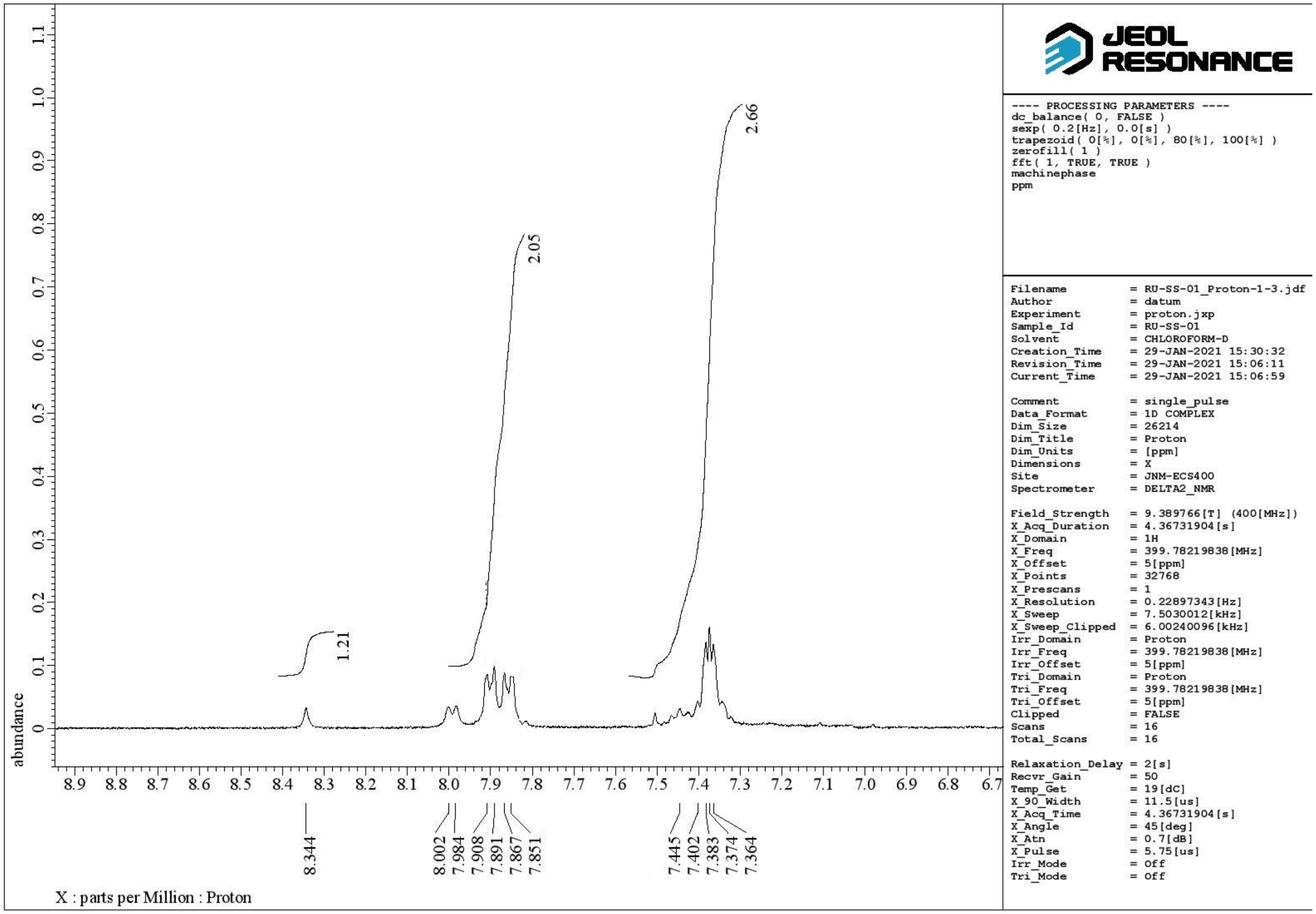

Figure 15a. ^{1}H NMR extended spectrum of (**2o**)

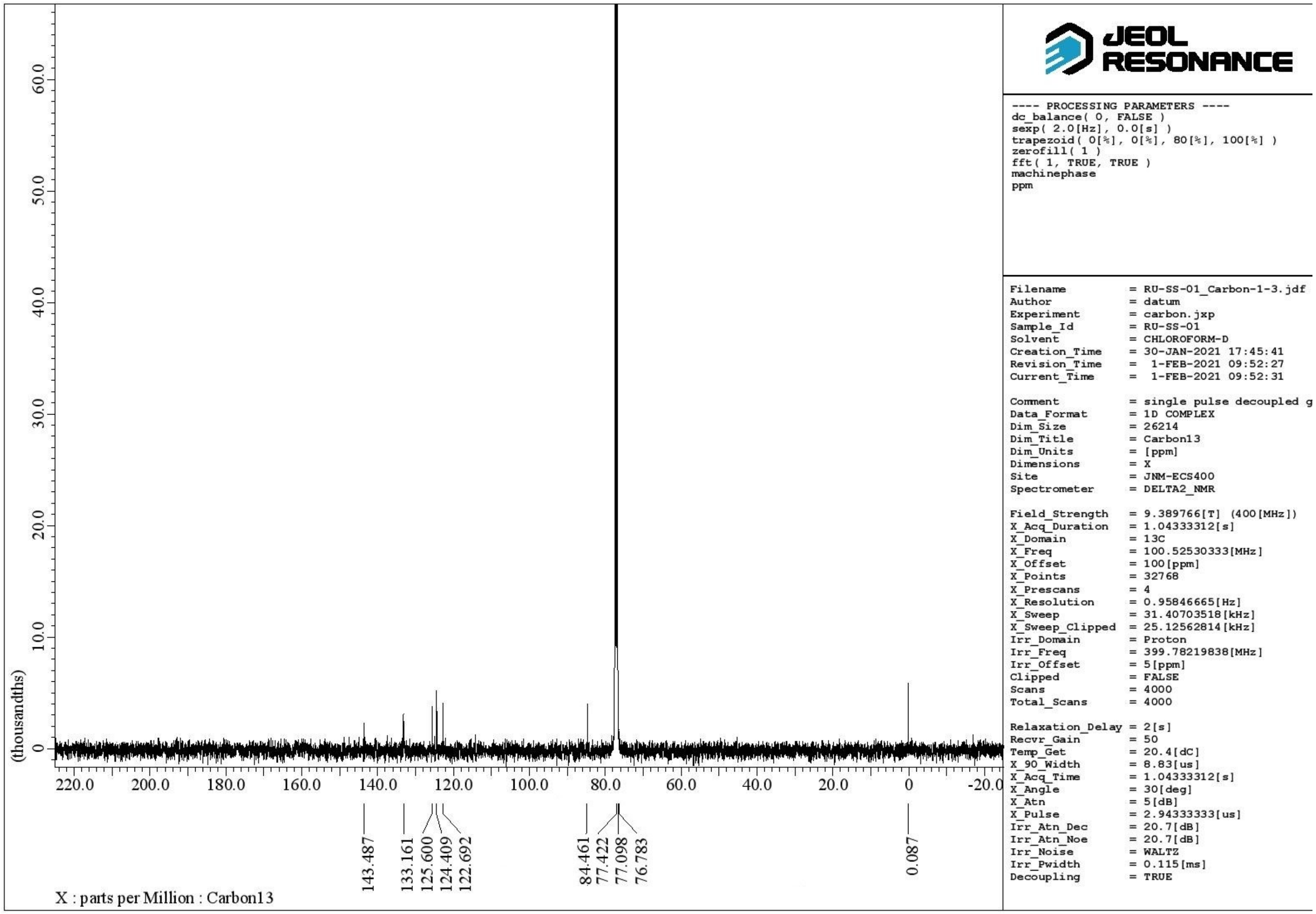

Figure 16. ^{13}C NMR spectrum of (**2o**)

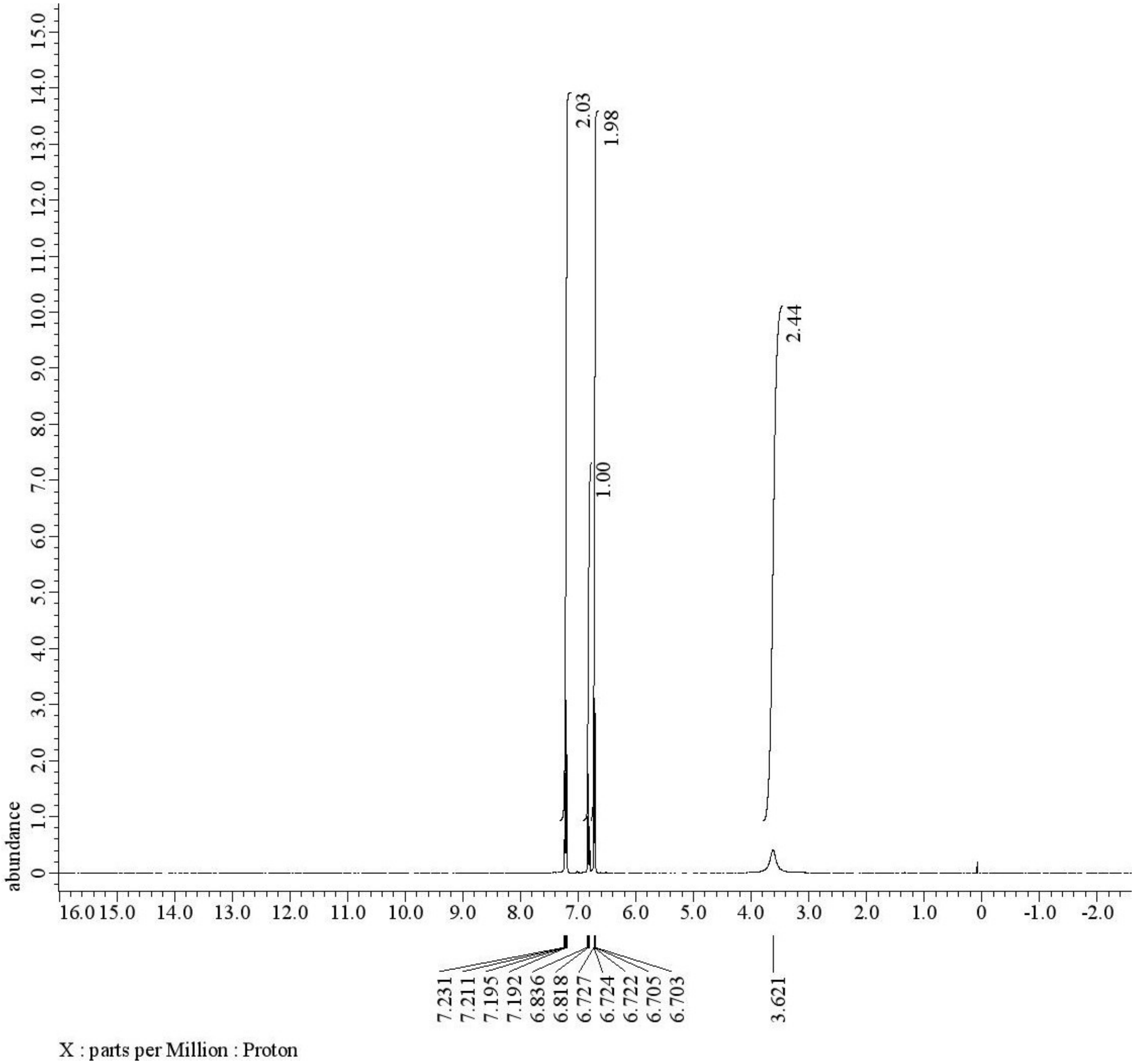

Figure 17. ^{1}H NMR spectrum of (**3a**)

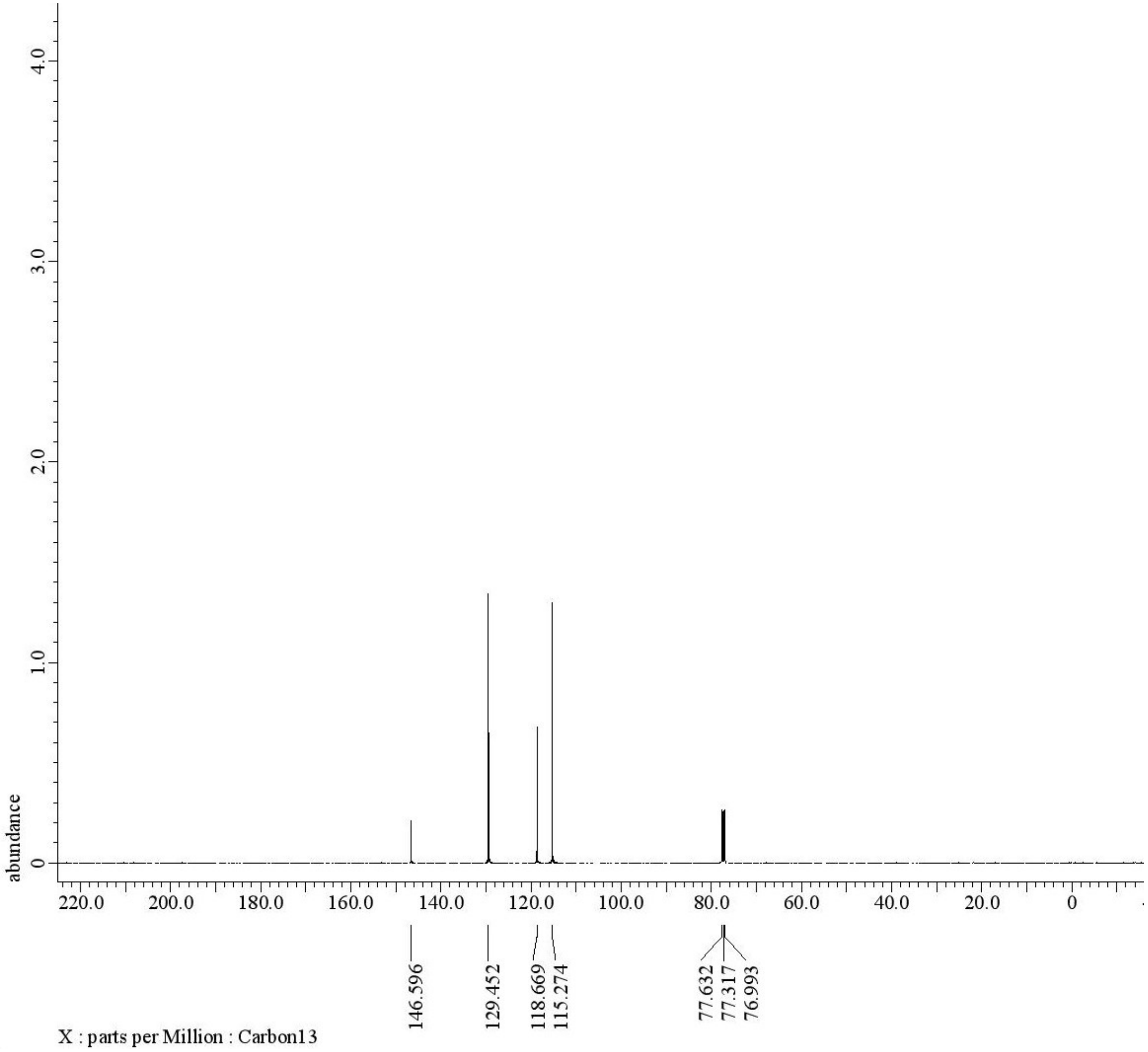

Figure 18. ^{13}C NMR spectrum of (**3a**)

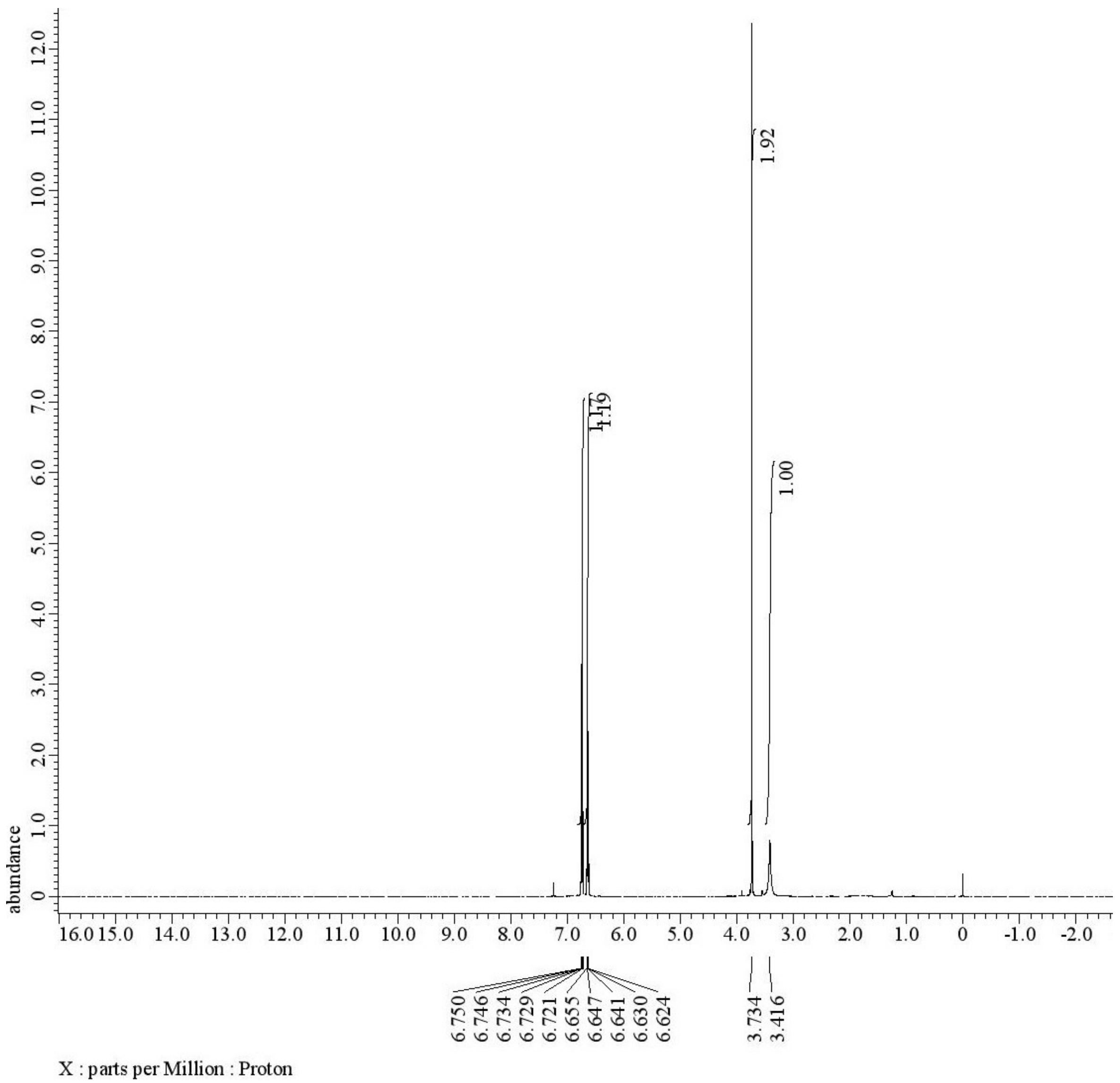

Figure 19. ^{1}H NMR spectrum of (**3c**)

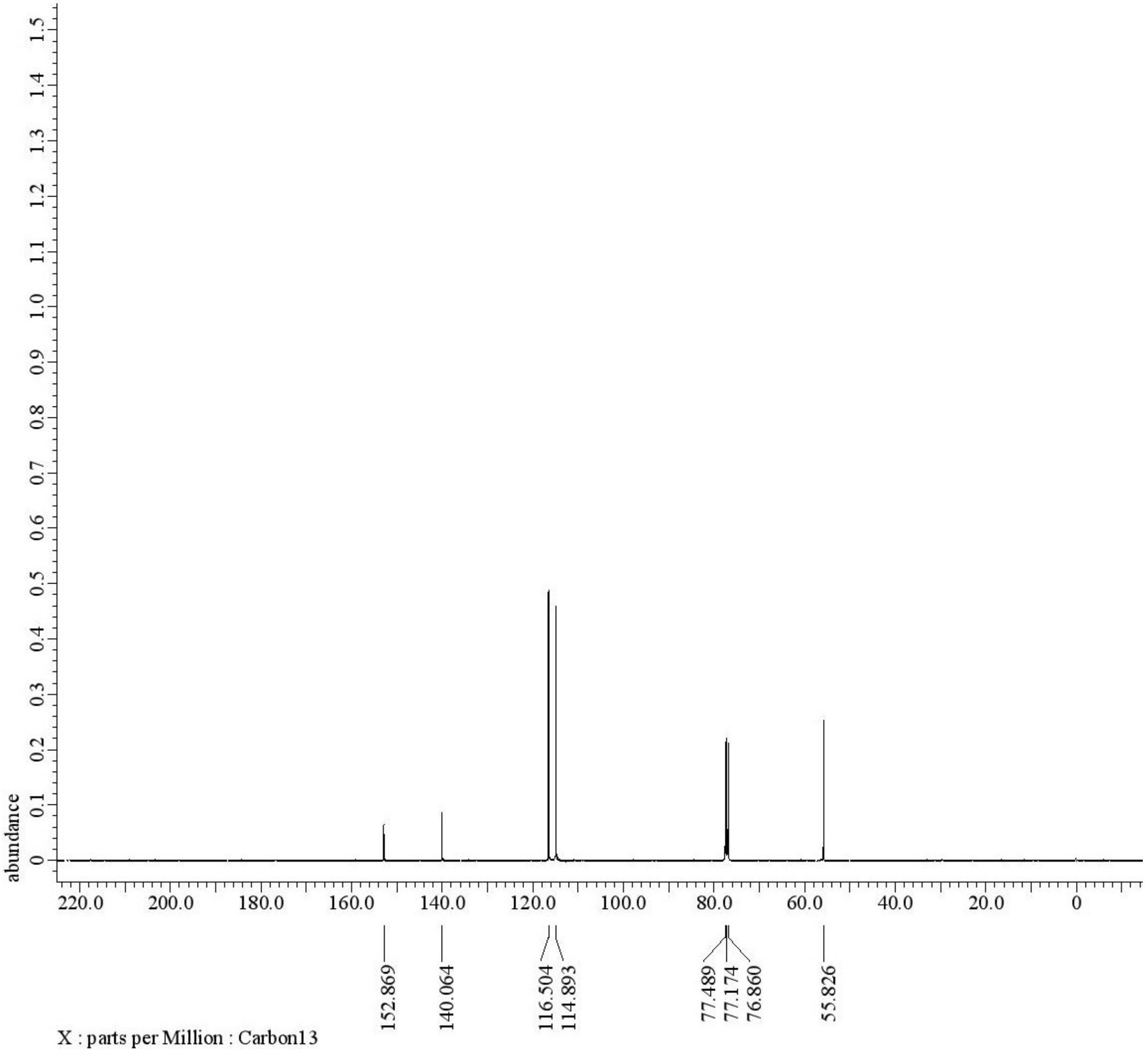

Figure 20. ^{13}C NMR spectrum of (**3c**)

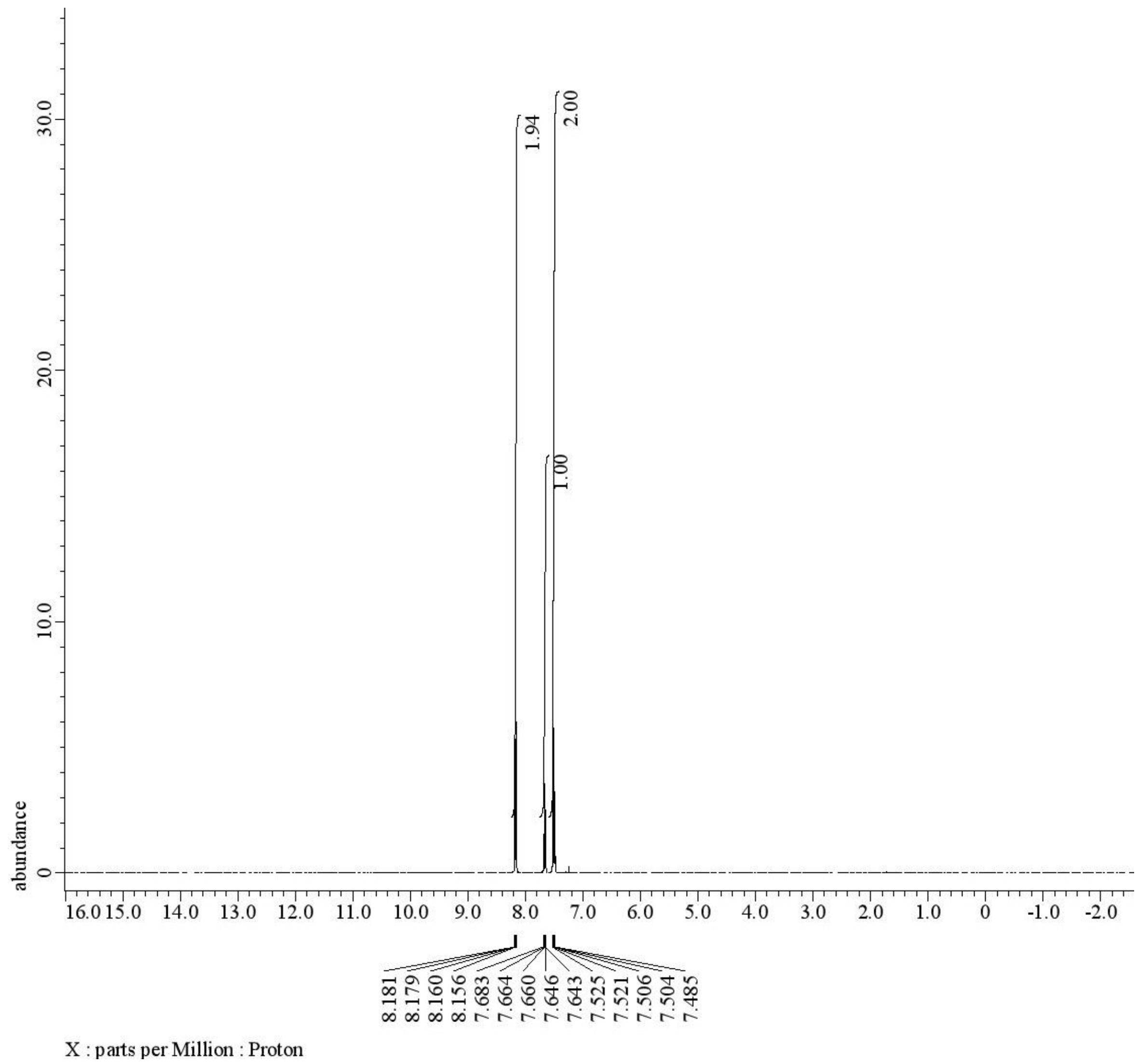

Figure 21. ^{1}H NMR spectrum of (**4a**)

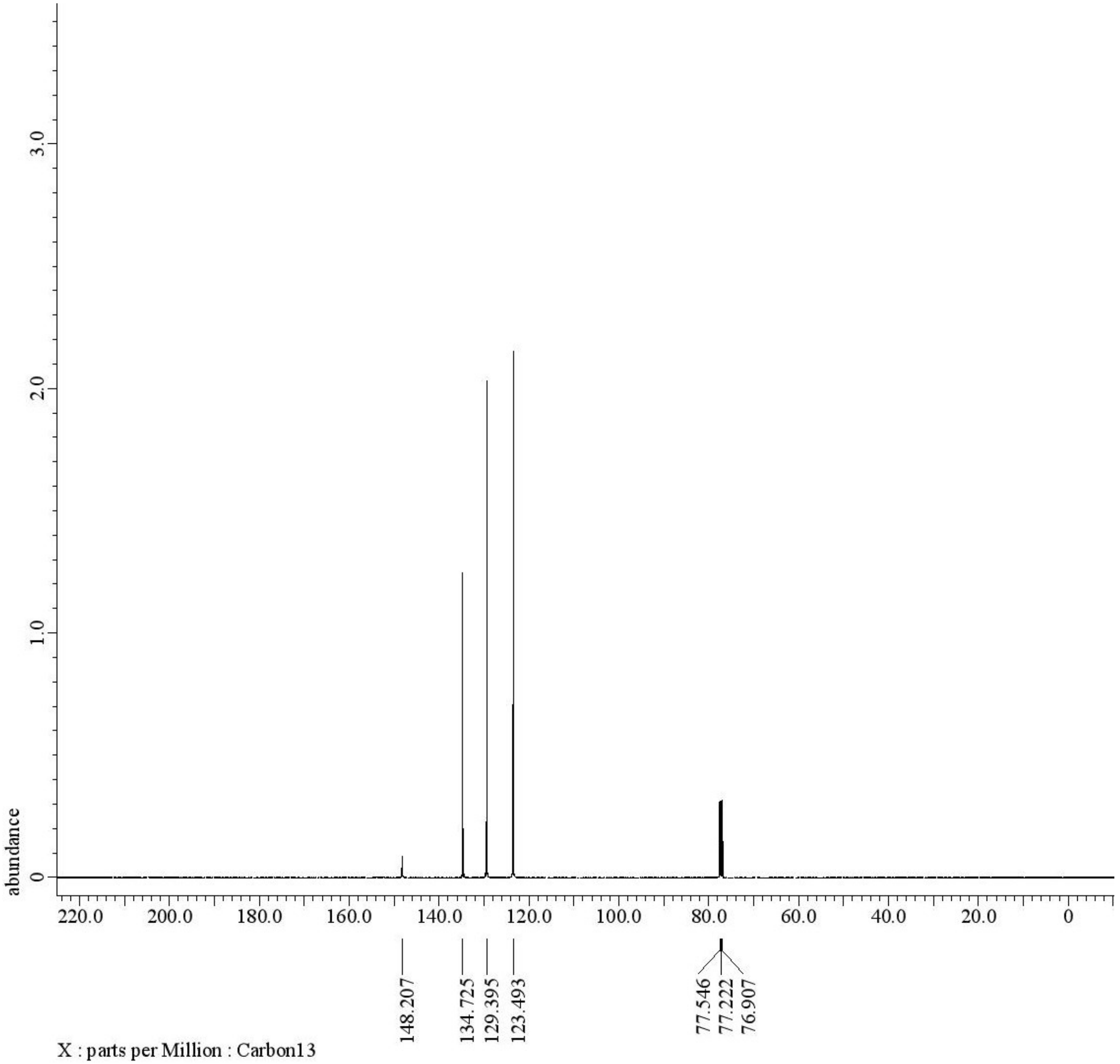

Figure 22. ^{13}C NMR spectrum of (**4a**)

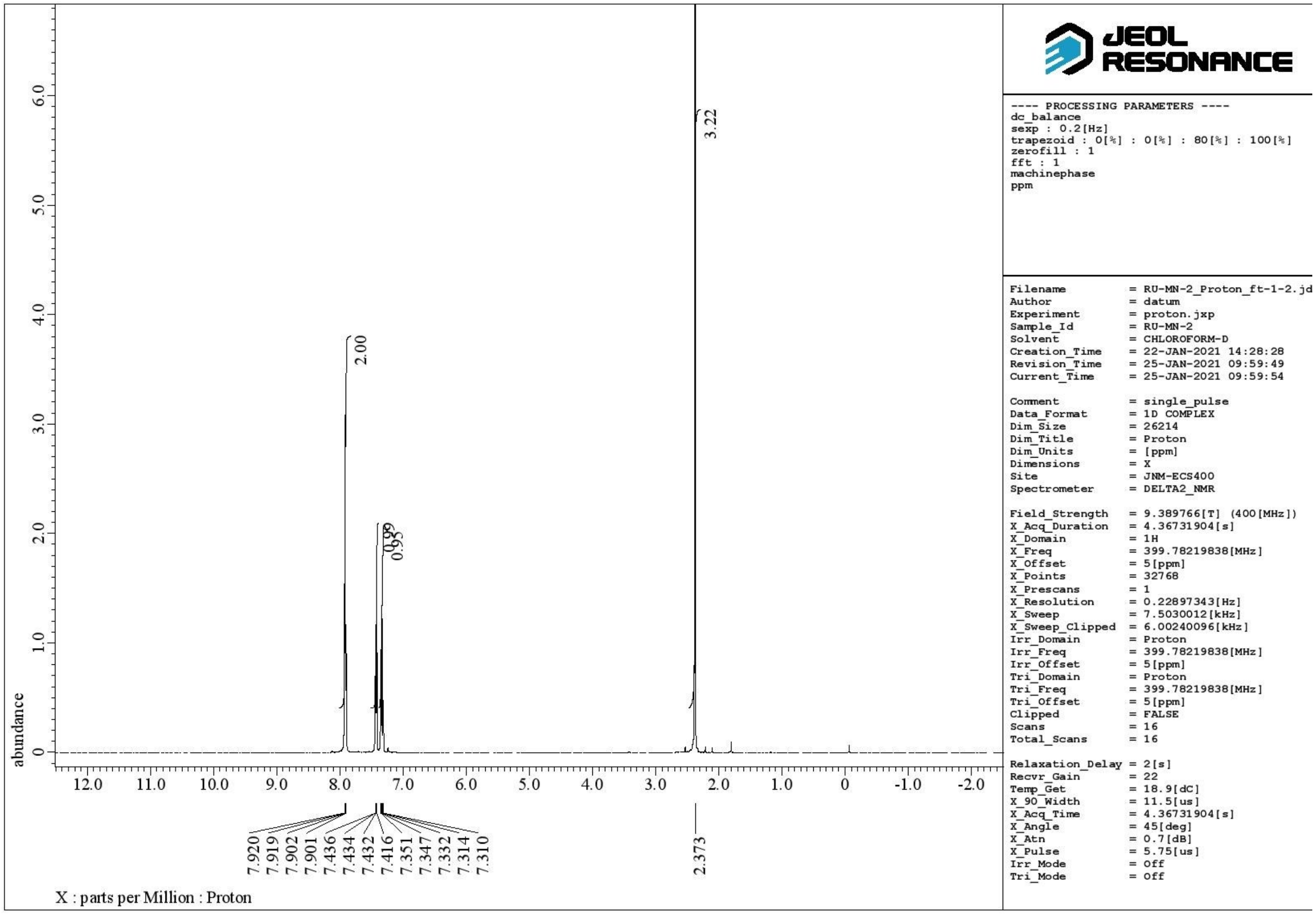

Figure 23. ^{1}H NMR spectrum of (**4c**)

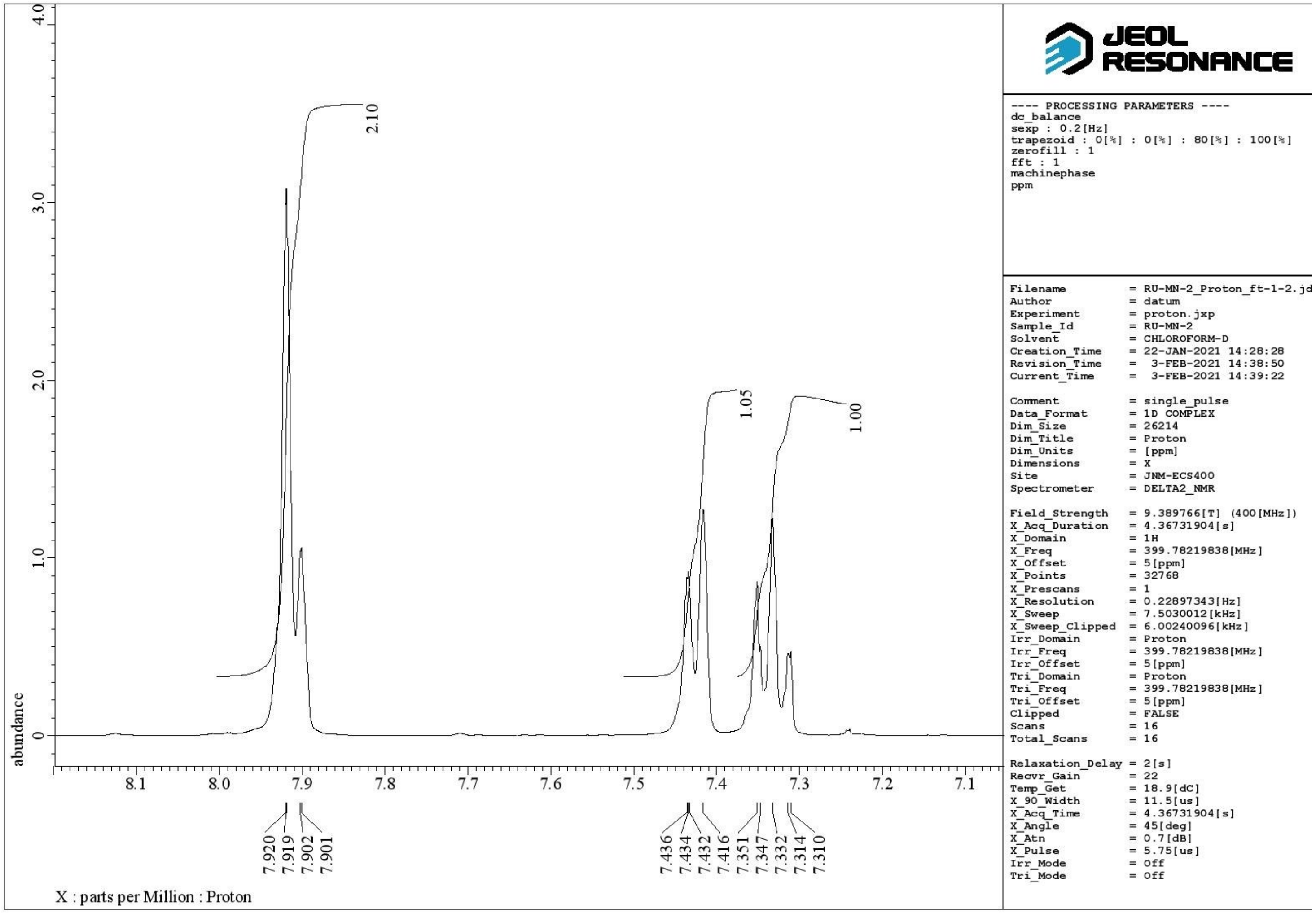

Figure 23a. ^{1}H NMR extended spectrum of (**4c**)

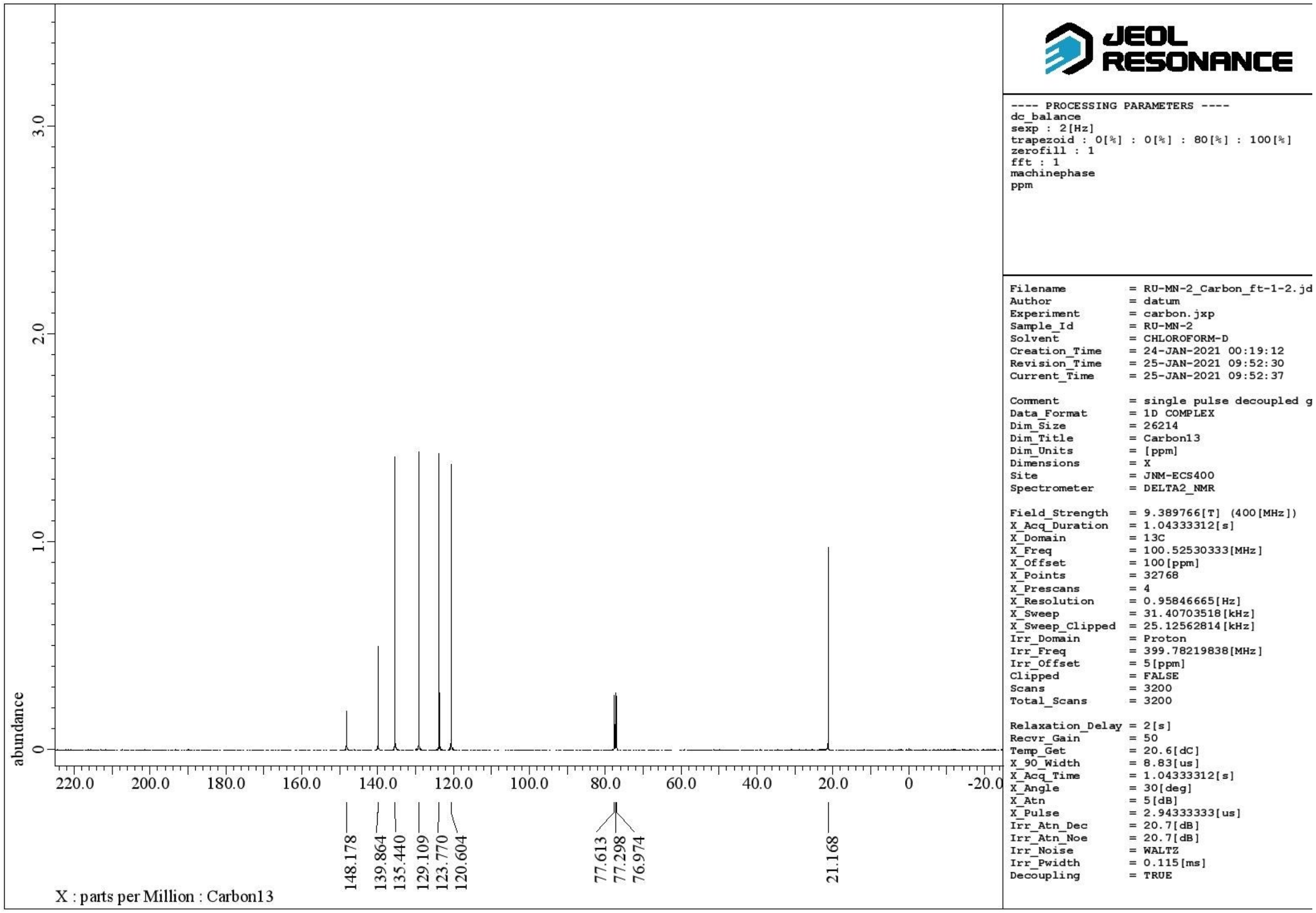

Figure 24. ^{13}C NMR spectrum of (**4c**)

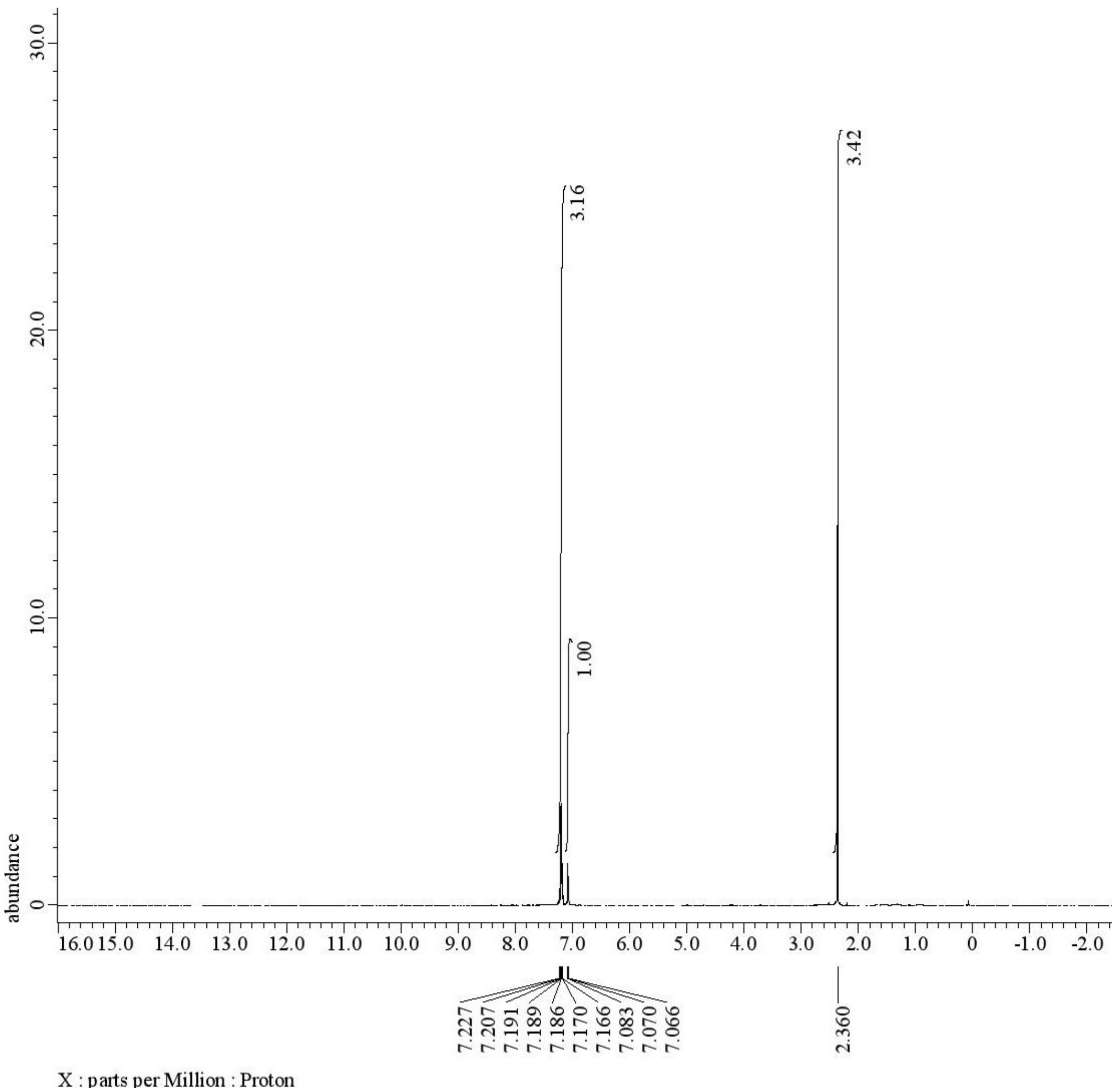

Figure 25. ^{1}H NMR spectrum of (**5c**)

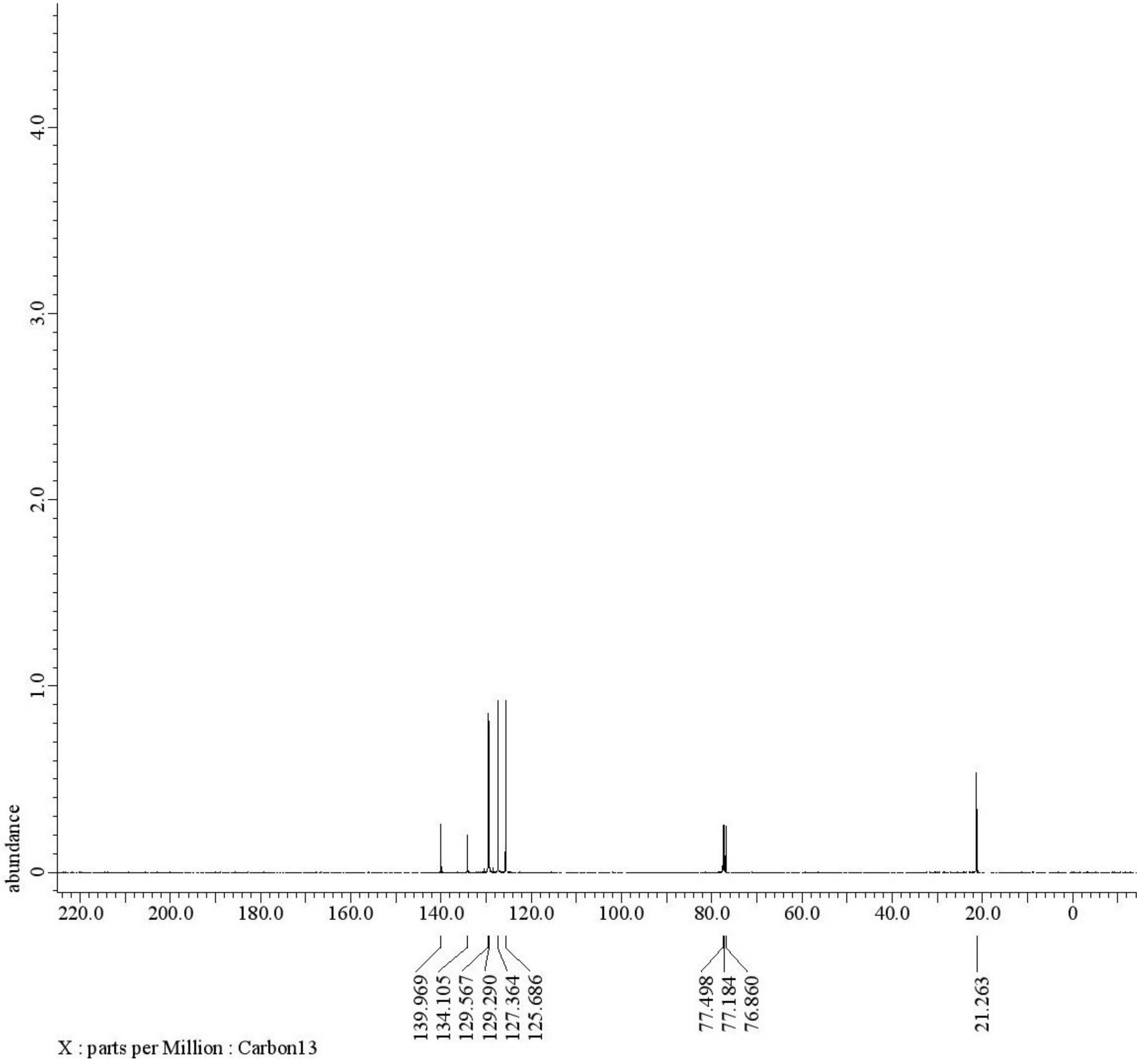

Figure 26. ^{13}C NMR spectrum of (**5c**)

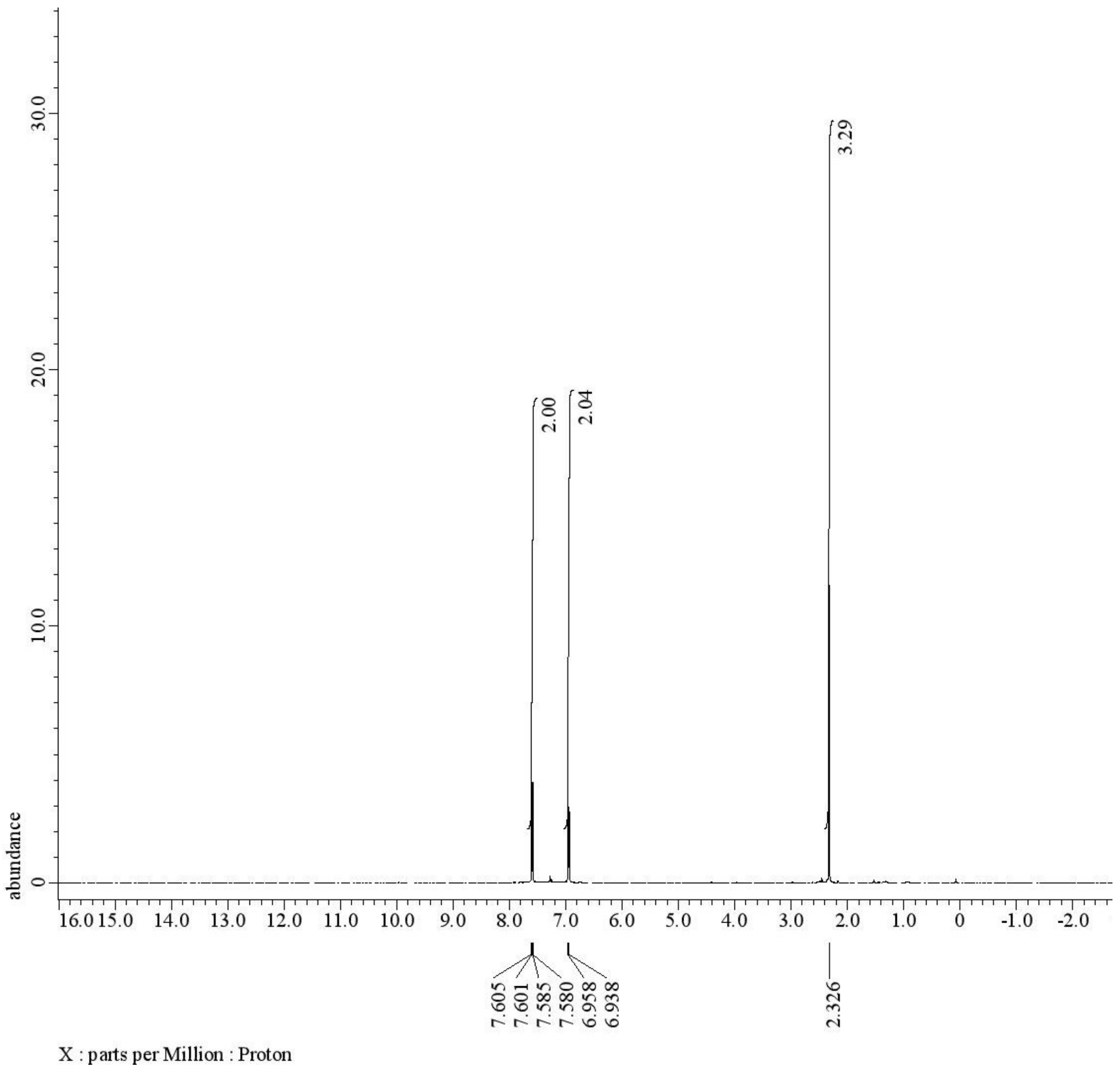

Figure 27. ^{1}H NMR spectrum of (**6b**)

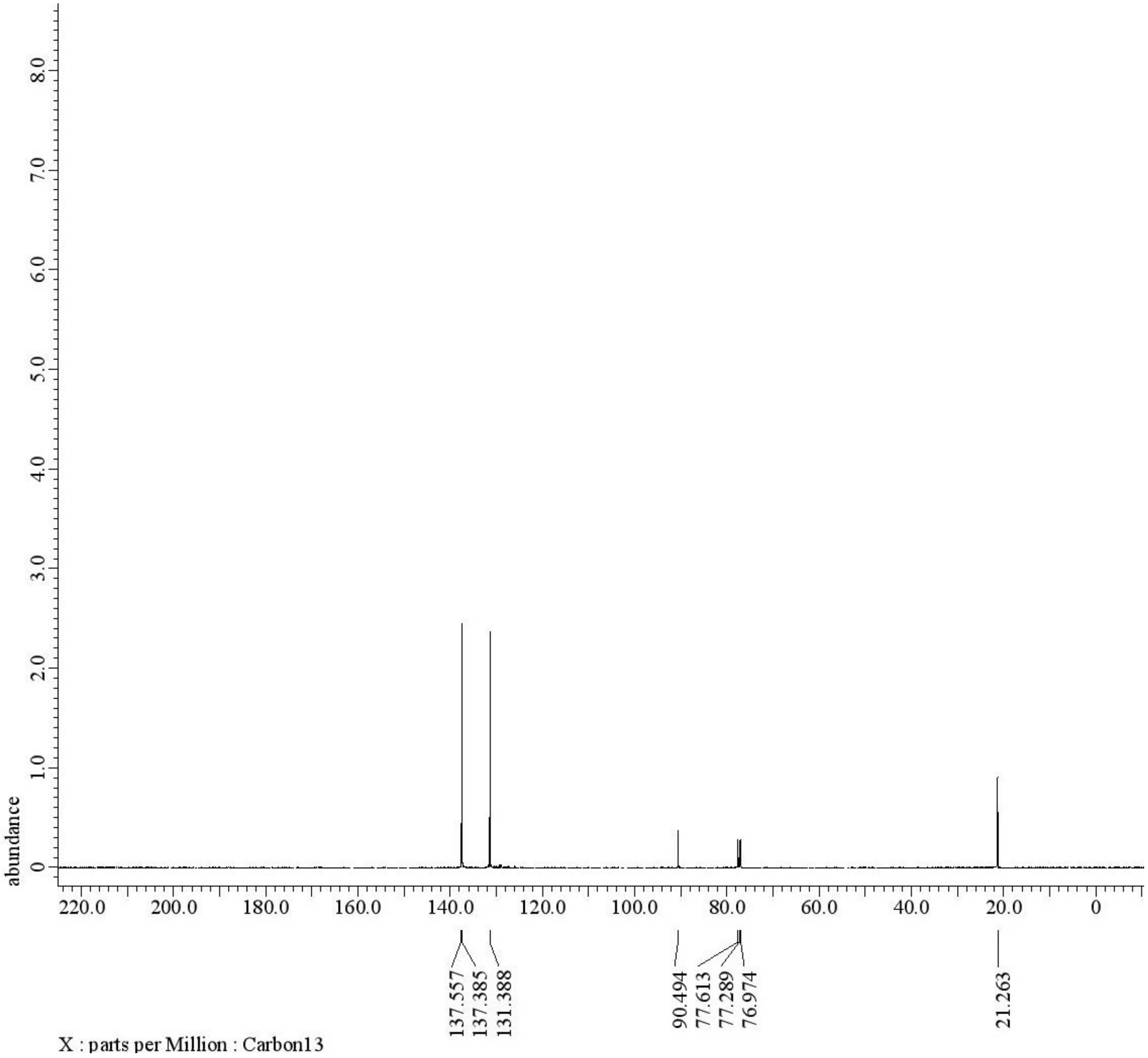

Figure 28. ^{13}C NMR spectrum of (**6b**)

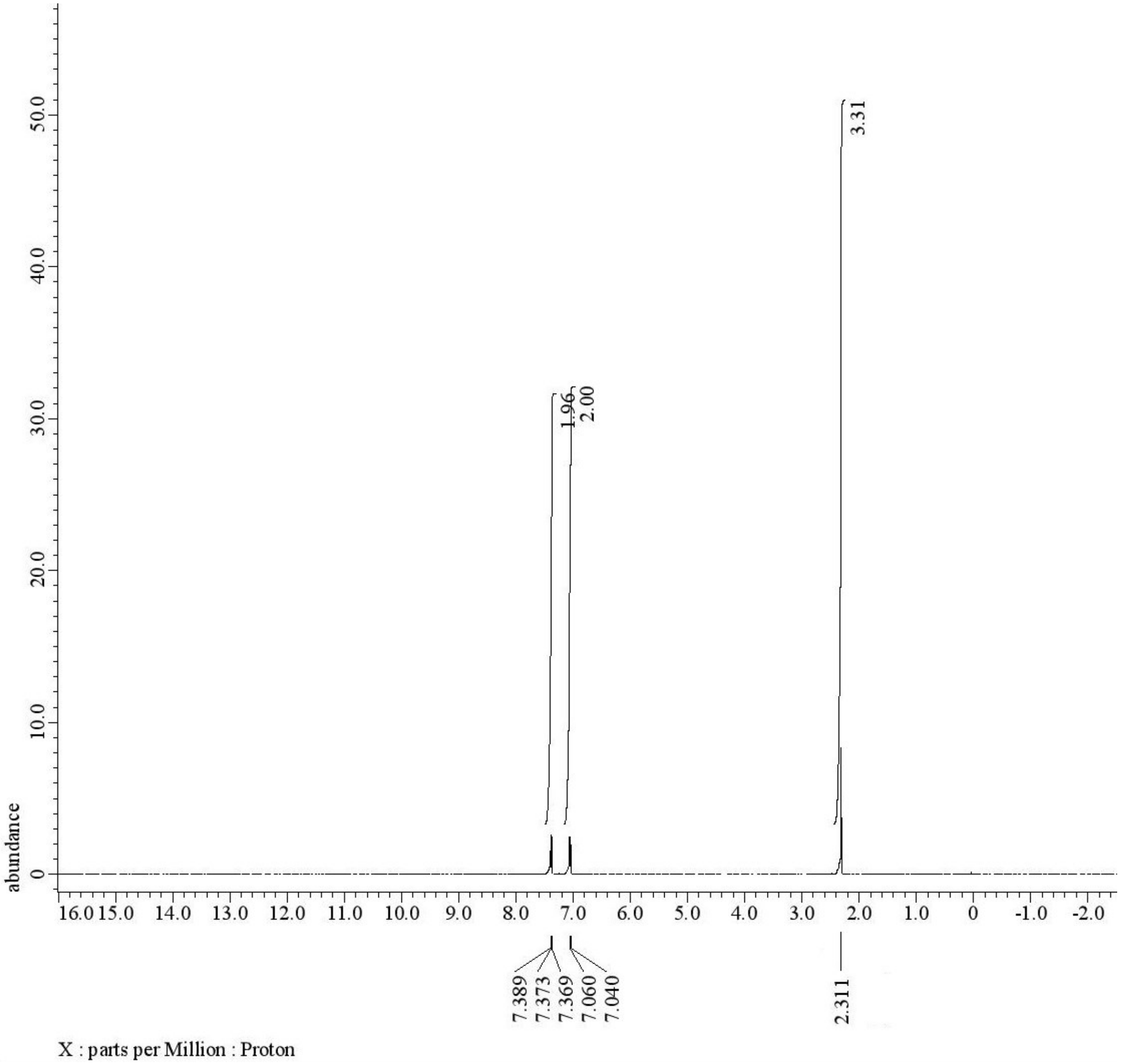

Figure 29. ^{1}H NMR spectrum of (**7b**)

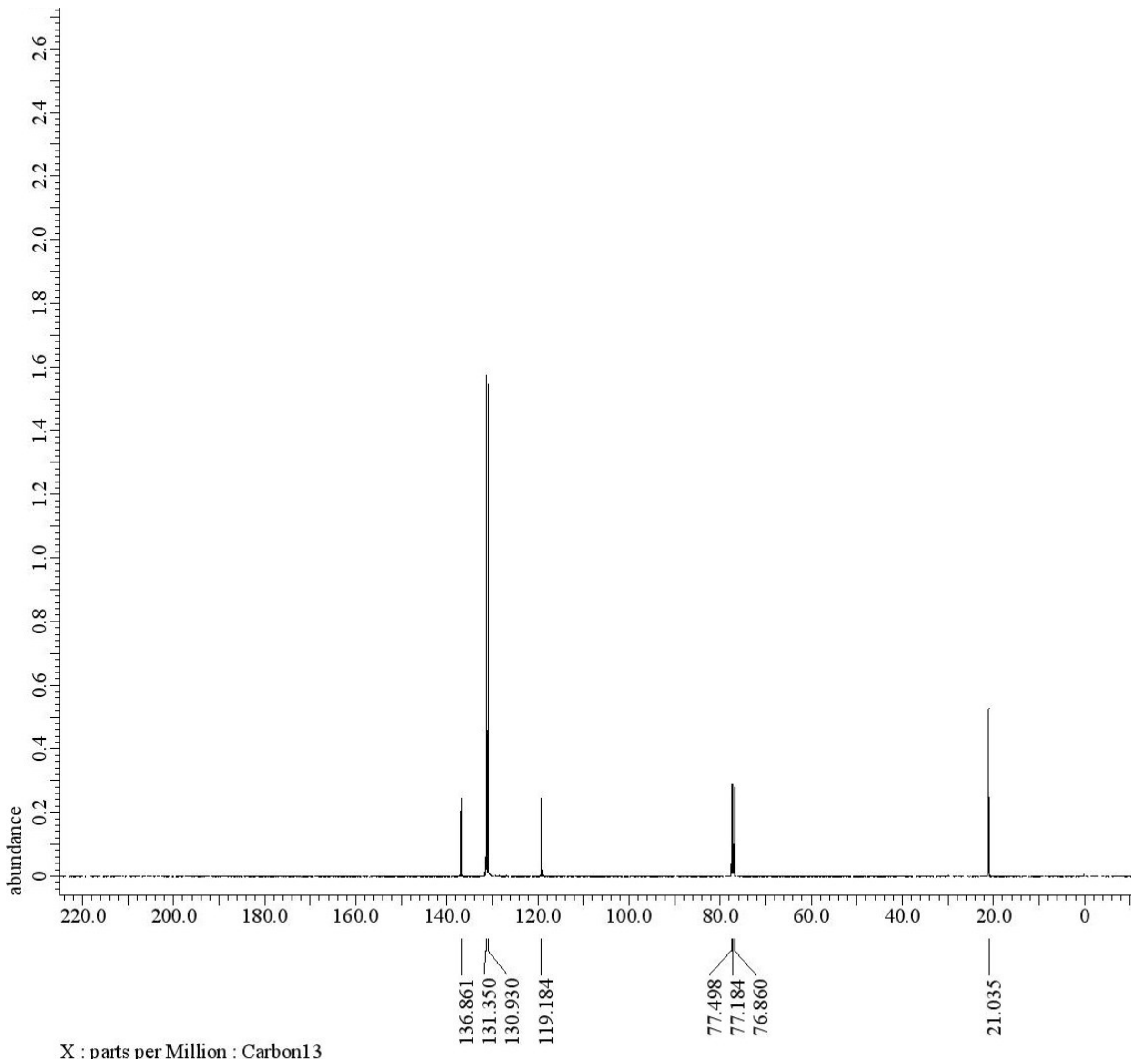

Figure 30. ^{13}C NMR spectrum of (**7b**)

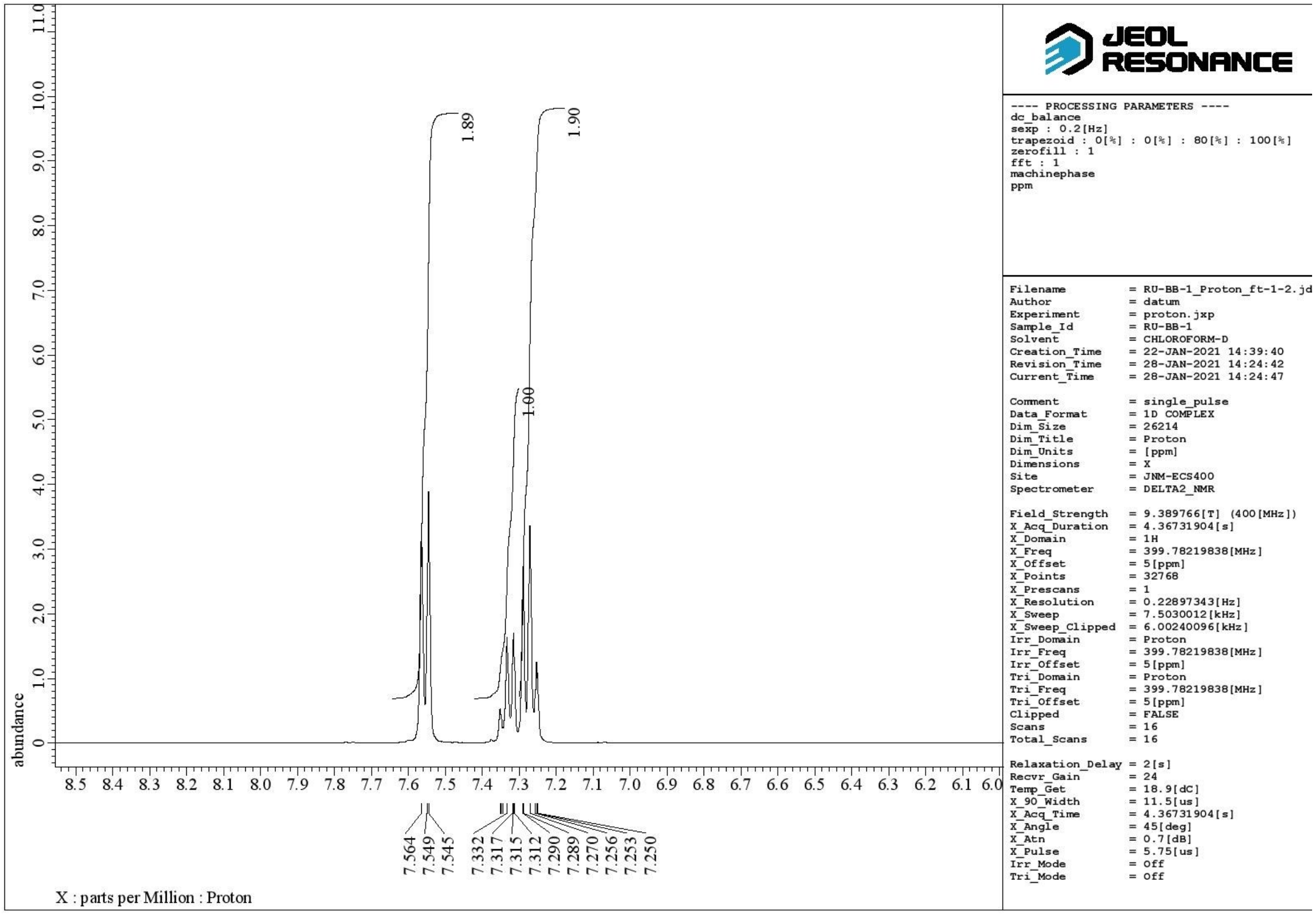

Figure 31. ^{1}H NMR spectrum of (**7c**)

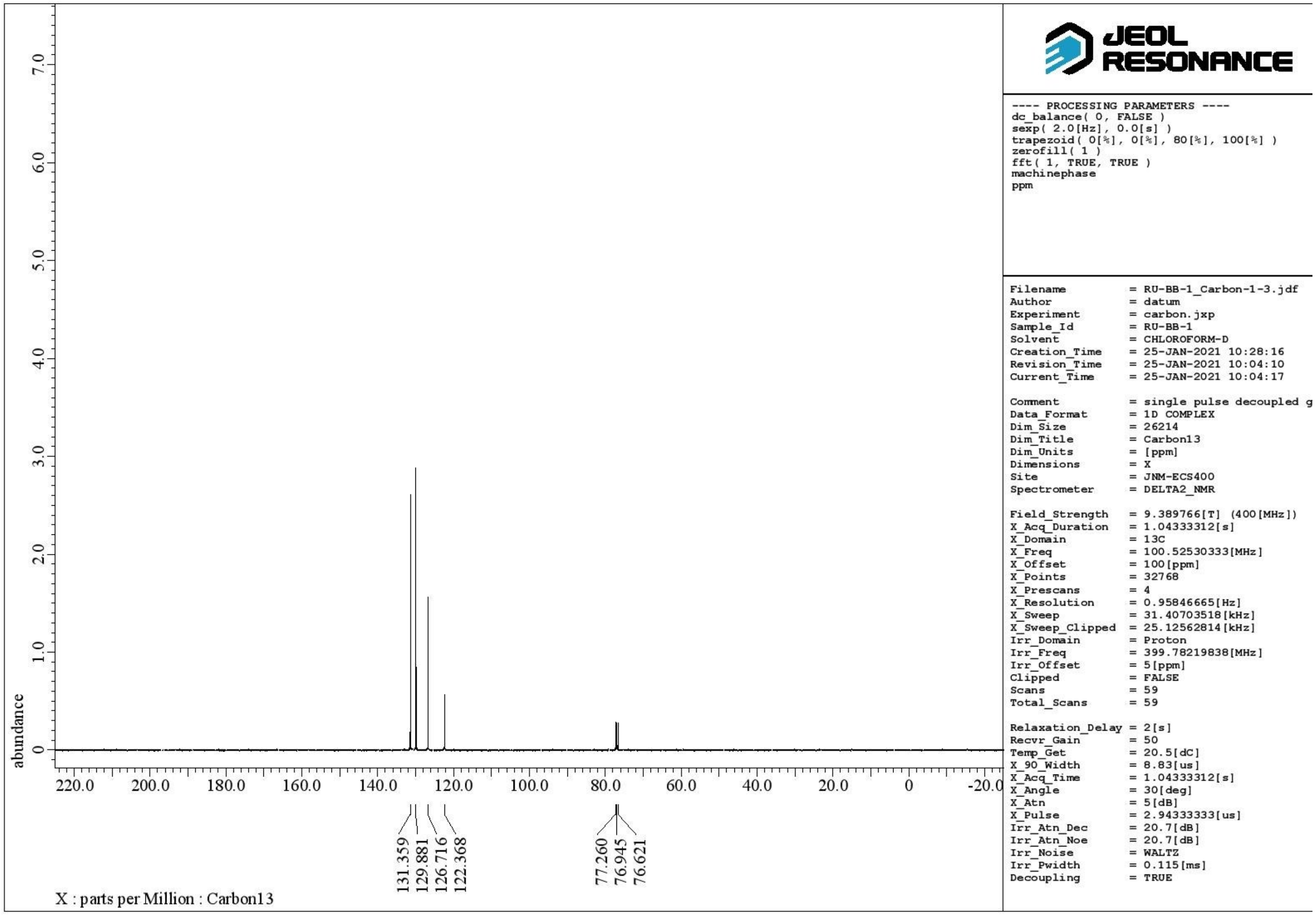

Figure 32. ^{13}C NMR spectrum of (**7c**)
